# Advancing Deuterium MRI to Track Human Cerebral Glucose Metabolism at 7 T: A Comparison of Glucose‐d_2_ and Glucose‐d_7_ Ingestion

**DOI:** 10.1002/nbm.70169

**Published:** 2025-11-03

**Authors:** Daniel J. Cocking, Robin A. Damion, Elizabeth J. Simpson, Dorothee P. Auer, Richard Bowtell

**Affiliations:** ^1^ Sir Peter Mansfield Imaging Centre University of Nottingham Nottingham UK; ^2^ School of Physics and Astronomy University of Nottingham Nottingham UK; ^3^ NIHR Nottingham Biomedical Research Centre/Nottingham Clinical Research Facilities Queen's Medical Centre Nottingham UK; ^4^ Mental Health and Clinical Neurosciences, School of Medicine University of Nottingham Nottingham UK; ^5^ School of Life Sciences University of Nottingham Nottingham UK

**Keywords:** deuterated glucose, DMI, glucose metabolism, glycolysis, lactate

## Abstract

Deuterium metabolic imaging (DMI) allows non‐invasive dynamic in vivo assessment of transport, uptake and metabolism of deuterated molecules. To date, DMI experiments in humans have involved ingestion of glucose‐d_2_ ([6,6’‐^2^H₂]glucose), where labelling of the sixth carbon facilitates ^2^H‐label transfer to pyruvate, then to lactate (Lac) via lactate dehydrogenase, or to glutamate and glutamine (Glx) via the tricarboxylic acid cycle. There are advantages to using glucose‐d_7_ ([1,2,3,4,5,6,6’‐^2^H₇]glucose) for DMI as this should yield larger signals from glucose and downstream metabolites, including deuterated water (HDO). Here, we evaluated DMI at 7 T following glucose‐d_7_ ingestion for monitoring glucose metabolism in the human brain. Results were compared to measurements using the same protocol but with oral glucose‐d_2_. Fifteen healthy volunteers participated in the study, which involved initial measurements at natural abundance, followed by 90 min of acquisition after ingestion of 0.75 g/kg glucose‐d_7_ (7 participants) or glucose‐d_2_ (8 participants). A visual stimulus was applied for 10 participants. Larger ^2^H signals were measured following glucose‐d_7_ ingestion, and whole‐brain signal ratios at times of 100 to 120 min after glucose‐d_7_ or glucose‐d_2_ ingestion for HDO, Glx and lactate (with potential contamination from lipid signals) were 1.8 ± 0.3, 1.7 ± 0.3 and 1.6 ± 0.3, respectively. At natural abundance, the SNR of the HDO signal in the CSI data was 14 ± 1. For both isotopologues, the glucose signal peaked ~80 min after ingestion, while Glx, lactate + lipid and HDO signals increased throughout the measurement period. Estimated cerebral concentrations of HDO were larger for glucose‐d_7_, but similar concentrations were found for glucose, Glx and lactate. No significant difference in signal or concentration between visually stimulated and unstimulated participants was found. These findings suggest that glucose‐d_7_ with DMI can facilitate non‐invasive in vivo assessment of metabolism in the human brain, with wide applications in experimental medicine and disease.

## Graphical abstract

Deuterium metabolic imaging (DMI) enables non‐invasive assessment of glucose metabolism in the human brain. This study compared the use of glucose‐d_7_ ([1,2,3,4,5,6,6’‐^2^H_7_]glucose) and glucose‐d_2_ ([6,6’‐^2^H_2_]glucose) as an ingested tracer in 15 healthy participants at 7 T. Glucose‐d_7_ provided larger deuterium signals for glucose, lactate, glutamate/glutamine (Glx) and deuterated water (HDO). Using glucose‐d_7_ in conjunction with DMI can facilitate the non‐invasive in vivo assessment of metabolic reprogramming in the human brain, with a wide range of applications in experimental medicine and disease.

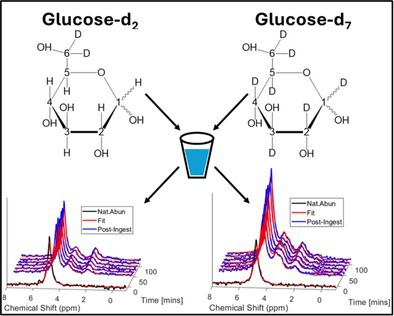

**Abbreviations**
BET: Brain extraction tool
BMI: Body mass index
CSF: Cerebrospinal fluid
CSI: Chemical shift images
DMI: Deuterium metabolic imaging
FSL: FMRIB Software Library
FAST: FMRIB's Automated Segmentation Tool
FDG PET: [^18^F]fluorodeoxyglucose positron emission tomography
FNIRT: FMRIB's Non‐linear Image Registration Tool
Glx: Glutamate and glutamine
Glucose‐d_2_
: [6,6’‐^2^H_2_]glucose
Glucose‐d_7_
: [1,2,3,4,5,6,6’‐^2^H_7_]glucose
GM: Grey matter
HDO: Deuterated water (semi‐heavy water)
Lac: Lactate
MNI: Montreal Neurological Institute‐Hospital
MPRAGE: Magnetisation‐prepared rapid gradient echo
MRSI: Magnetic resonance spectroscopic imaging
NA: Natural abundance
ROI: Region of interest
SNR: Signal‐to‐noise ratio
TCA: Tricarboxylic acid
WM: White matter

## 1 Introduction

Deuterium metabolic imaging (DMI) combines magnetic resonance spectroscopic imaging (MRSI) with the use of deuterium (^2^H) as a non‐radioactive hydrogen isotope tracer to allow non‐invasive dynamic in vivo assessment of transport, uptake and metabolism of deuterated molecules, such as glucose [1]. In DMI experiments using labelled glucose, the sixth carbon position is usually labelled ([6,6’‐^2^H_2_]glucose) to facilitate ^2^H‐label transfer to pyruvate and then to lactate (Lac) via lactate dehydrogenase, or to glutamate and glutamine (Glx) via the tricarboxylic acid (TCA) cycle [1, 2]. De Feyter et al. successfully detected glucose uptake and downstream metabolites in the human brain following oral ingestion of [6,6’‐^2^H_2_]glucose (glucose‐d_2_) and demonstrated a substantially increased lactate/Glx ratio in brain cancer as a marker of metabolic reprogramming that leads to increased glycolysis and downregulated oxidative phosphorylation, known as the Warburg effect [3]. Importantly, the capability to detect downstream metabolites of deuterated glucose means that DMI has the potential to overcome limitations of fluorodeoxyglucose positron emission tomography (FDG PET) by allowing the altered uptake of glucose and its consumption through distinct metabolic pathways to be disentangled.

A key limitation of DMI is its low intrinsic signal‐to‐noise ratio (SNR) due to the 6.5‐fold lower gyromagnetic ratio of ^2^H compared to ^1^H (6.54 vs. 42.57 MHz/T). However, as a spin‐1 nucleus, ^2^H has a quadrupolar moment which leads to shorter T_1_ relaxation times. This allows the use of shorter repetition times and hence faster signal averaging. The low natural abundance of deuterium is advantageous as it leads to low background signal and thus easier interpretation of label enrichment. To exploit the full potential of this promising technique, ongoing research efforts aim to overcome the low SNR of DMI through optimised signal detection techniques [4] and increased signal generation via use of ultra‐high‐field MR (7 T and higher [5, 6, 7, 8]). To date, DMI voxel volumes in human studies have often been 8 mL [9, 10], although smaller volumes of 2–3 mL [7, 11] have been reported and more recently 0.75 mL [12] volume was achieved at 7 T using concentric ring trajectories and de‐noising. Improved spatial resolution can also be realised through indirect detection using ^1^H MRSI [5, 8, 11, 13, 14, 15, 16], with the additional benefit of not requiring deuterium‐specific hardware, but at the expense of requiring more complex acquisition and analysis to separate label effects from large background signals.

An alternative approach to signal enhancement would be to directly increase the deuterium signal through ingestion of a higher dose of labelled glucose. However, this amount of glucose may not be well tolerated or safe, nor efficient in increasing the amount of labelled cerebral glucose uptake, due to the expected homeostatic control of postprandial hyperglycaemia and blood–brain glucose transport. The capacity of intestinal absorption is unlikely to be saturated by higher glucose load [17], but blood brain glucose transport is downregulated in the postprandial state compared to fasted [18]. Interestingly, a recent small pilot study comparing glucose detection in the brain with DMI after ingestion of 0.25, 0.5 and 0.75 g/kg of deuterated glucose suggested no gain beyond 0.5 g/kg [19]. Introducing a larger number of deuterium labels per glucose molecule is an attractive alternative approach that provides a desirable SNR boost without the disadvantages of excessive glucose loading. Substitution of hydrogen atoms in the hydroxyl groups of glucose is not useful for metabolic applications as such ^2^H labels will exchange rapidly with any ^1^H atoms in water. However, labelling all seven carbon‐bonded atoms that are less labile, i.e., [1,2,3,4,5,6,6’‐^2^H_7_]‐glucose (glucose‐d_7_), may be useful. Compared with glucose‐d_2_, the deuterium spectrum of glucose‐d_7_ contains more components, many of which overlap due to the broad linewidths, potentially producing a net gain in SNR. The deuterium atoms in the C1 and C6 positions (in the absence of label‐loss) are transferred to lactate or Glx molecules [20], producing an expected gain of 3/2 in those signals in DMI. In addition, the four remaining deuterium labels in the positions C2–C5 of glucose‐d_7_ are transferred directly or indirectly to water during glycolysis and therefore contribute to an increased HDO signal [21, 22, 23]. Figure 5 in Mahar et al. [22] provides a detailed depiction of the pathways for label‐loss from glucose‐d_7_ (and by inference also glucose‐d_2_) into HDO. Glucose‐d_7_ has previously been used for ^2^H‐NMR‐based measurements of glucose metabolism in mice [24, 25], excised rat hearts [26], lipogenesis in mice [27], cell cultures [21, 23] and DMI measurements in mice [20, 28] and rats [22], but has not yet been used in human studies.

In this study we evaluated the use of glucose‐d_7_ ingestion in conjunction with DMI measurement for monitoring in vivo glucose metabolism in the human brain. The results were compared to measurements made using the same protocol but with oral intake of glucose‐d_2_. Fifteen healthy volunteers participated in the study which involved initial measurements at natural abundance, followed by 90 min of data acquisition after ingestion of 0.75 g per kg body weight of glucose‐d_7_ (7 participants) or glucose‐d_2_ (8 participants). To boost SNR, measurements were made using a 7‐T scanner. These measurements have been used to characterise the spatio‐temporal patterns of glucose uptake and enrichment of Glx, lactate+lipids and HDO, allowing us to propose an optimised protocol and to report normative metabolic ratios for biomedical and clinical applications.

## 2 Experimental

### 2.1 Human Participants

Ethical approval was received from the Faculty of Medicine and Health Sciences Research Ethics Committee (Ref: FMHS 306–0621) at the University of Nottingham to recruit healthy volunteers for this study. Informed consent was obtained from all participants meeting the inclusion/exclusion criteria: age between 18 and 60 years; BMI < 25 kg/m^2^ (or less than 27 kg/m^2^ for males, if their waist circumference was less than 94 cm); normal heart rate and blood pressure; a blood glucose concentration < 7.8 mM (finger‐prick test); no significant medical conditions or issues related to safety in the 7 T MR scanner. In total, 15 participants were recruited. Eight participants ingested glucose‐d_2_ and seven ingested glucose‐d_7_ (see Table 1 for summary statistics of the cohorts).

**TABLE 1:** Summary statistics for cohorts with respect to the glucose isotopologue and whether a visual stimulus was applied (VS) or no stimulus was applied (NS).

Cohort	Weight (kg)	BMI (kg/m^2^)	Age (years)	*n*
Glucose‐d_2_, VS	65 ± 5	22 ± 3	29 ± 12	6
Glucose‐d_2_, NS	56 ± 14	19 ± 4	22 ± 2	2
Glucose‐d_2_, VS + NS	63 ± 9	22 ± 3	27 ± 11	8
Glucose‐d_7_, VS	71 ± 6	22 ± 1	33 ± 13	4
Glucose‐d_7_, NS	63 ± 20	20 ± 5	25 ± 5	3
Glucose‐d_7_, VS + NS	67 ± 15	21 ± 3	30 ± 11	7

*Note:* VS + NS implies the combined VS and NS cohorts. *n* is the total number of participants in each cohort.

### 2.2 Study Protocol

Participants were asked to fast for at least 8 h prior to commencing the scanning session. To check that participants were in a fasted state, a blood glucose concentration test (finger‐prick) was administered at the start of the session, and this was deemed to be acceptable if the blood glucose concentration was < 5.6 mM.

Scanning for each participant was split into two parts: (i) a first scanning session, lasting approximately 20 min, which was carried out before ingestion of the glucose drink to gather natural abundance measurements for quantification and normalisation, and (ii) a second 90‐min scanning session carried out after the glucose drink had been ingested to track the temporal variation of ^2^H signals from the glucose and its metabolic products. The protocol is schematically visualised in Figure S1 in Supplementary Material.

In the first session, two ^1^H scans were acquired (survey and MPRAGE anatomical images), followed by three deuterium scans (a non‐selective spectrum, a slice‐selective spectrum and a 3D CSI).

After the first scanning session, the participant exited the scanner and was given the glucose drink. The drink consisted of 0.75 g/kg (body weight) of either glucose‐d_2_ or glucose‐d_7_ powder (between 31 and 68 g) dissolved in 250 mL of water at room temperature. The glucose powder was purchased from CK Isotopes Ltd. (microbiological/pyrogen‐tested product) and Merck Life Science UK Ltd. (endotoxin‐tested product). The participants were allowed to consume this drink in their own time, and on average took 3.25 min (1–11 min range). The second session of scanning commenced at times ranging from 2 to 31 min after ingestion, with times varying between participants to provide measurements over a larger range of times post‐glucose ingestion.

In the second session, the two ^1^H scans were repeated, followed by five or six consecutive repeats of the three different deuterium acquisitions. If necessary, participants took a comfort break, exiting the scanner for a short period before re‐entering. This break was followed by a repeat of the ^1^H scans before continuing with the deuterium scans. For 10 of the 15 participants (6 who ingested glucose‐d_2_ and 4 who ingested glucose‐d_7_), a visual stimulus was given (details in Table 1) to explore whether such a stimulus affected the measured metabolite concentrations.

### 2.3 MR System and Scan Details

Scanning was performed on a 7 T Achieva scanner (Philips Healthcare), operating at 45.8 MHz for ^2^H. A 26.4 cm inner‐diameter, dual‐tuned ^1^H/^2^H birdcage RF coil (Rapid Biomedical) was used for the acquisition of deuterium measurements and anatomical ^1^H images. The 4‐kW broadband RF amplifier normally used on the scanner for measurement of signals from ^1^H and other nuclei operates in the frequency range 70–300 MHz. For these experiments, we therefore connected a 4‐kW amplifier (CPC, New York USA) that operated at lower frequencies (10–130 MHz) spanning the ^2^H resonance frequency.

MPRAGE ^1^H scans were acquired with the following parameters: FOV = 224 x 224 x 140 mm^3^, 1.4 mm isotropic voxels, TR = 7.1 ms, TE = 2.6 ms, TI = 1050 ms, flip angle = 1° and a scan duration of 353 s. Non‐localised ^2^H spectra were acquired using 16 averages, TR = 1000 ms, TE = 1.1 ms, flip angle = 90°, bandwidth = 3000 Hz, 2048 samples, with a scan duration of 17 s. Slice‐selective ^2^H spectra were acquired from a 2‐cm‐thick axial slice positioned over the lateral ventricles, using 128 averages, TR = 1000 ms, TE = 1.9 ms, flip angle = 90°, bandwidth = 3000 Hz, 2048 samples, having a scan duration of 129 s. 3D ^2^H chemical shift images (CSI) covering the whole brain were acquired using 6 acquisition‐weighted [29] averages, FOV = 180 × 180 × 120 mm^3^, 15 mm isotropic voxels, TR = 230 ms, TE = 2.4 ms, flip angle = 62° (truncated sinc excitation of 3.27 ms duration), bandwidth = 1200 Hz, 256 samples, with a scan duration of 670 s.

### 2.4 Image and Spectral Processing

^1^H MPRAGE images were converted to NIfTI format using MRIcroGL (www.nitrc.org), bias‐field corrected using FSL's FAST [30], and then brain‐extracted using FSL's BET [31]. The MNI‐152 brain template with 2 mm isotropic voxels (distributed with FSL [32]) was non‐linearly registered to the corrected MPRAGE image to obtain the warp‐field using FSL's FNIRT [33]. The warp‐field was used to non‐linearly register the MNI‐space partial‐volume segmentation maps of the frontal and occipital cortices to the MPRAGE space. The frontal and occipital masks overlaid onto the MNI‐152 brain template are shown in Figure S2 in the Supplementary Material. Finally, FSL's FAST was used to segment the corrected MPRAGE image to form partial volume maps for cerebrospinal fluid (CSF), grey matter (GM) and white matter (WM). The CSF mask was manually edited to include only the left and right ventricles. The maps were then binarised to obtain region of interest (ROI) masks.

Each CSI data set was denoised using a Tucker decomposition [34] which reduced the original data matrix from [*N*_FID_, *N*_AP_, *N*_RL_, *N*_slice_] = [256, 12, 12, 8] to [64, 6, 6, 4], where [*N*_FID_, *N*_AP_, *N*_RL_, *N*_slice_] are the dimensions corresponding to the sampled FID, anterior–posterior, right–left and slice (foot‐head) image directions, respectively. This compression is similar to that used in previous DMI [35, 36] and ^13^C studies [37]. All data matrices were fully reconstructed, using this compressed core matrix, to the original resolution before continuing with fitting. FIDs were then fitted using an adapted version of the OXSA‐AMARES MATLAB toolbox [38, 39], which requires prior knowledge of each metabolite's signals. The ^2^H chemical shift values for glucose (both anomers), Glx and lactate were assumed to be the same as the ^1^H chemical shifts [40] and were implemented in the fitting as relative shifts to the water peak [41]. A minimum reporting standard in MRS (MRS in MRS) table is completed in the Supplementary Material Table S12. No lipid removal has been applied here [42] and therefore the signal strength of the 1.3 ppm resonance is reported as lactate+lipids (Lac+Lip) as we cannot rule out lipid contamination. We have however carried out concentration estimates based on analyzing this signal using the effective label number and T_1_‐values for lactate. We report these values as estimated lactate concentrations but note that they may be affected by contaminating lipid signals. In previous DMI studies that have used glucose‐d_2_, the glucose signal has generally been fitted as a single peak at 3.8 ppm, since the chemical shift differences between the four resonance lines (two deuterium labels, two glucose anomers) are usually not discernible due to the relatively broad linewidths. However, this is not the case for glucose‐d_7_ which has a larger number of spectral lines spanning a wider range of chemical shifts [40]. The spectral analysis of glucose‐d_7_ data therefore accounted for the contribution from each deuterium label for both anomers and, for consistency, this approach was also used when analyzing the glucose‐d_2_ data. The glucose peaks were fitted assuming a common amplitude for each peak of a given anomer but allowing the anomers to have different amplitudes. All glucose peaks were assumed to have the same phase, other than the peaks from the C1 position which were fitted with a different phase due to the large difference in chemical shift from the other glucose peaks. The linewidths of the 14 peaks from both glucose anomers shared the same value. For glucose‐d_2_, the prior knowledge was the same as for glucose‐d_7_ but restricted to the four relevant peaks from ^2^H at the C6 position. This process produces a fitted glucose signal amplitude which is 7/2 times larger for a given concentration of glucose‐d_7_ compared to the same concentration of glucose‐d_2_. For HDO, Glx and lactate, only single components were assumed, with independent amplitudes, phases and linewidths. Details of the fitting parameters can be found in the Supplementary Material, Table S3.

The fitted amplitude and phase of each metabolite peak at each voxel were converted to complex amplitudes and interpolated to the same resolution as the MPRAGE image. These maps were then averaged over the whole‐brain, occipital cortex and frontal cortex ROIs (using the binarised segmentation maps) to obtain ROI‐averaged amplitudes for each metabolite for each CSI, which provided amplitudes as a function of time relative to glucose ingestion. The masks in the MNI space were checked to ensure they did not overlap with the lateral ventricles before and after registration.

### 2.5 Concentration Calculations

Concentrations $C^{m}$ for each metabolite m were determined using the equation

(1) $$C^{m}=\frac{A^{m}}{\kappa E^{m}N^{m}}$$

where $A^{m}$ is the amplitude of the metabolite's FID, $N^{m}$ is the number of effective deuterium labels per metabolite molecule, $E^{m}$ is the attenuation factor given by

(2) $$E^{m}=\frac{1-e^{-T_{R}/T_{1}^{m}}}{1-e^{-T_{R}/T_{1}^{m}}\left(\cos\theta \right)}$$

where $T_{1}^{m}$ is the longitudinal relaxation time of the metabolite, $T_{R}$ is the repetition time, θ is the applied flip‐angle, and κ is a scaling constant. This constant is calculated for each ROI using the average water signal amplitude measured within that ROI before loading, and applying Equation 1 with an estimate of the natural abundance concentration calculated assuming an isotopic percentage for deuterium of 0.0156% [43], a concentration of pure water at 55.4 M and an estimate of the percentage of water depending on the mix of tissue‐types in the ROI. CSF, cortical grey matter (GM) and white matter (WM) were assumed to be 99%, 84% and 69% water [44]. The percentages of tissue‐types in the ROIs were estimated via segmentations of the MNI‐152 brain template and the MNI atlas for the occipital and frontal cortices. A summary of the calculations can be found in Table S4 of the Supplementary Material.

Once κ was calculated for each ROI, metabolite concentrations were calculated via Equation 1, with knowledge of the deuterium label numbers, $N^{m}$. The effective number of deuterium labels depends on whether glucose‐d_2_ or glucose‐d_7_ is ingested and, for Glx and lactate, also depends on label‐loss [26, 45]. To account for label‐loss for glucose‐d_2_, it was assumed that the effective numbers of labels per molecule were 1, 2, 0.6 and 0.85, respectively, for HDO, glucose, Glx and lactate. The values for Glx and lactate are calculated from the label‐loss data of de Graaf et al. [45], assuming that glutamine (Gln) and glutamate (Glu) are present in approximately the ratio Glu/Gln = 2 [40, 46], and also takes into account that each glucose molecule produces two pyruvate molecules but that only one of them is labelled. We assumed that the data of de Graaf et al. [45] for glucose‐d_2_ was also applicable to glucose‐d_7_. The effective numbers of labels were therefore assumed to be 1, 7, 0.9 and 1.28, respectively, for HDO, glucose, Glx and lactate, where the values for Glx and lactate have been scaled by 3/2 because, when using glucose‐d_7_, the two pyruvate molecules produced by glycolysis are expected to contain a total of three deuterium atoms in the absence of label‐loss.

Longitudinal relaxation times for glucose, Glx and lactate were assumed to be 67, 139 and 297 ms, respectively [1], independent of ROI, number of deuterium labels and whether glucose‐d_2_ or glucose‐d_7_ was the metabolic precursor. For water (HDO), the assumed T_1_‐relaxation times were 510, 320 and 290 ms for CSF, grey matter and white matter, respectively [47]. These values were used to estimate effective relaxation times for HDO based on the relative proportions of different tissue types contained in each ROI (Table S5 of the Supplementary Material).

### 2.6 Analysis of Metabolites

Dynamic profiles of metabolite enrichment were estimated for HDO, glucose, Glx and lactate by averaging temporal data across individual participants at the ROI level. Metabolite amplitude maps, averaged over temporal acquisitions for each participant, were also produced to visualise spatial distributions at the voxel level. Metabolite amplitudes resulting from the use of glucose‐d_7_ and glucose‐d_2_ were compared using participant‐averaged data by forming the ratio of a given metabolite derived from ingestion of the two glucose isotopologues. Time‐dependent ratios of metabolite concentrations to the sum of Glx and lactate concentrations, and direct comparisons of lactate against Glx concentrations—to index glucose uptake as well as overall and relative glycolytic versus oxidative metabolism—were derived for the whole brain, frontal and occipital ROIs, for both glucose‐d_7_ and glucose‐d_2_ measurements.

### 2.7 Statistics

Two‐sample, two‐tailed t‐tests were conducted to test whether mean Glx and lactate concentrations within an 80–100 min time window were different between the frontal and occipital cortex ROIs. The *p* values were calculated via MATLAB's *ttest2* function (MATLAB Version: 9.14.0.2674353 (R2023a) Update 7).

## 3 Results

**FIGURE 1:**
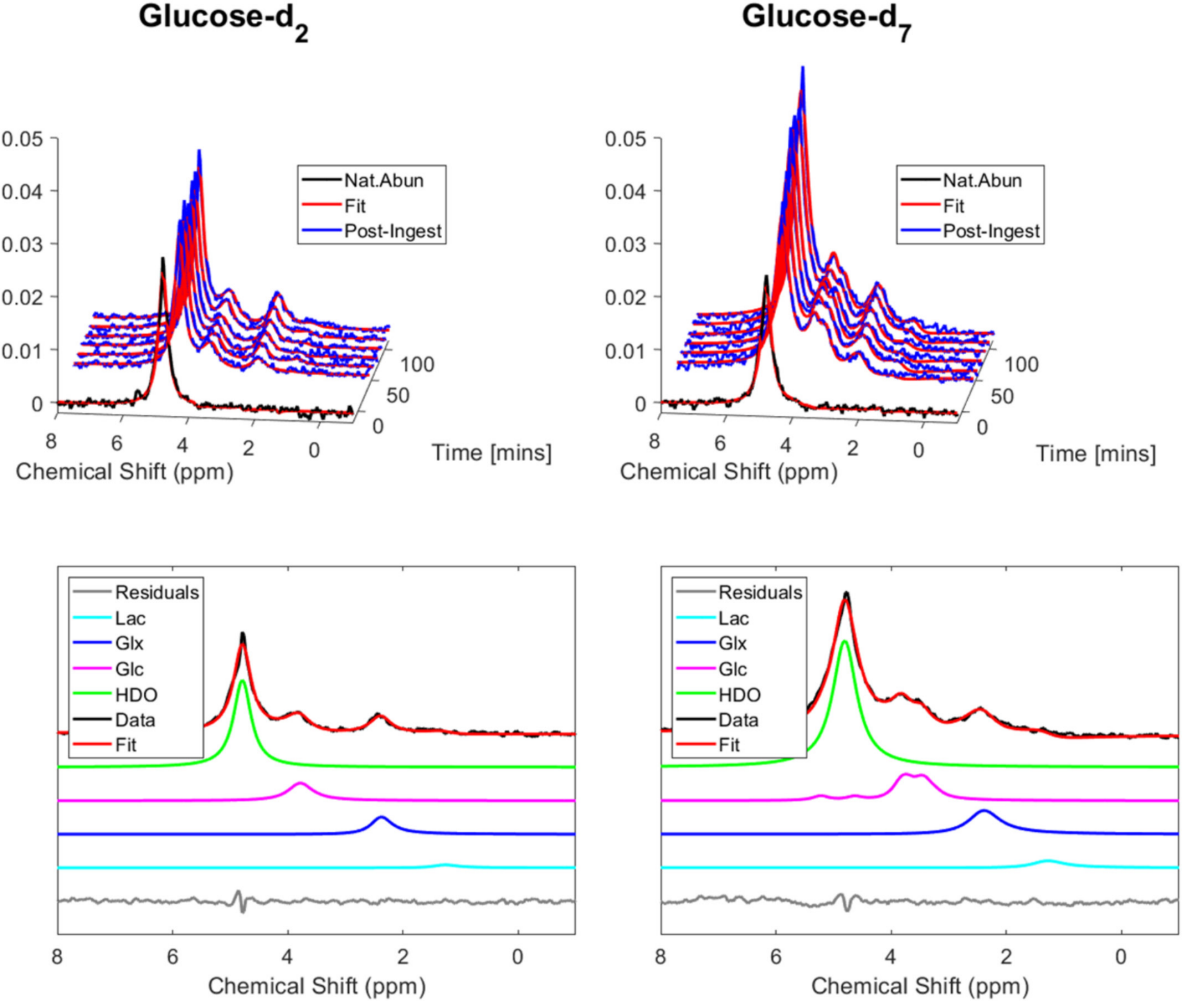
Spectra acquired from a 2 cm axial slice sited over the lateral ventricles, at natural abundance (top panels, black line) and after ingestion (blue lines) of glucose‐d_2_ (left) and glucose‐d_7_ (right), with fits to the spectra (red lines). Fits of spectra acquired approximately 100 min after glucose ingestion are shown in the bottom panels, along with fitted metabolite contributions and the residuals. These data are from different individual subjects.

**FIGURE 2:**
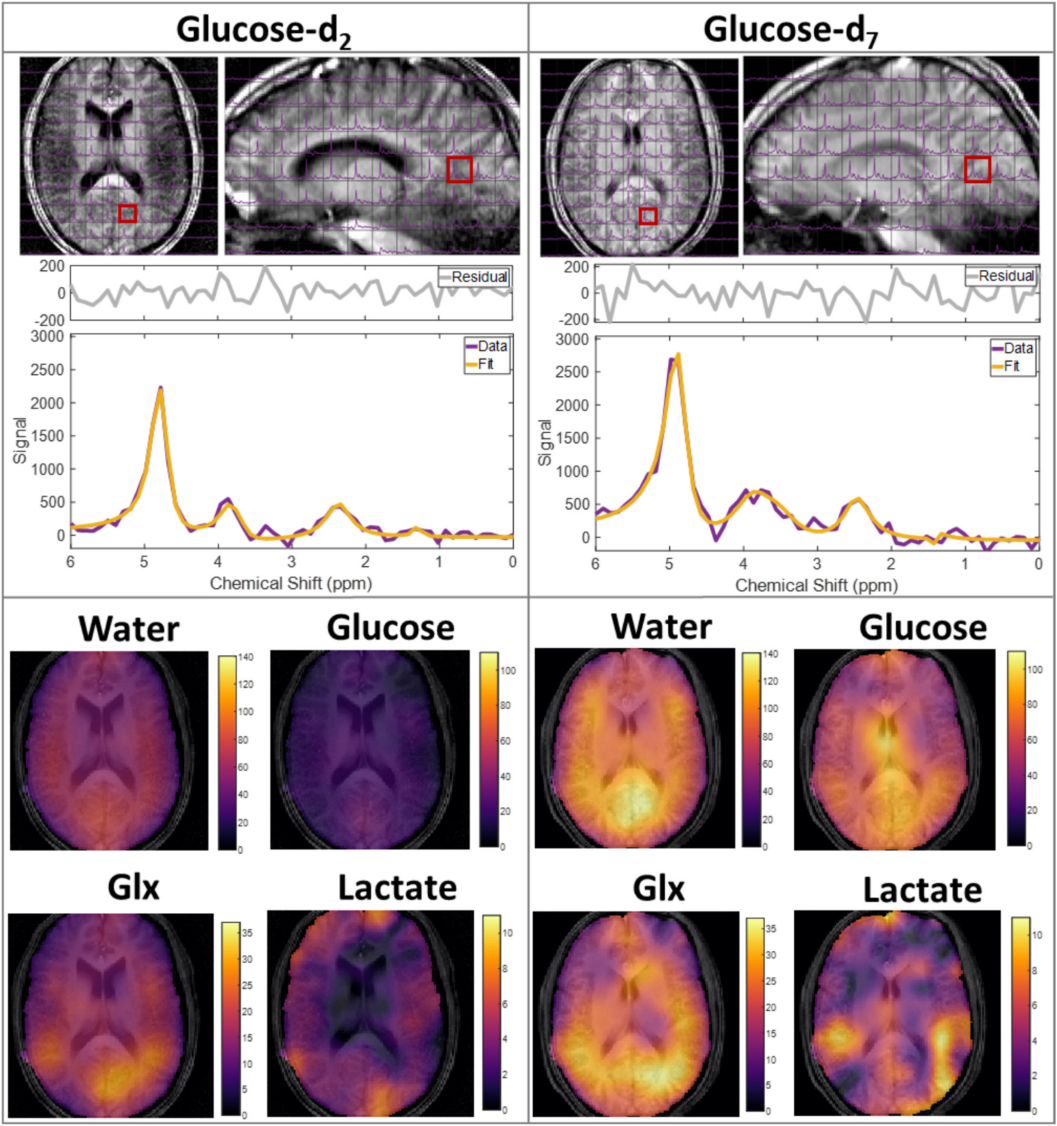
CSI data (purple) acquired after ingestion of glucose‐d_2_ (left) and glucose‐d_7_ (right) overlaid onto anatomical ^1^H MPRAGE images shown axially and sagittally. The CSI data is averaged over all measurements acquired at times of 50 min or more after ingestion of the glucose. Spectra from highlighted voxels (red) are shown below, with fits overlaid in yellow, along with the residual from the fits. Fitted amplitude maps for each metabolite are also shown in the lower panels, interpolated to the same resolution as the MPRAGE images. The data shown here is shown from two separate participants.

Representative spectra (Figure 1) show the natural abundance HDO signal before ingestion and the signals derived from the labelled glucose post‐ingestion, with higher signal amplitudes from glucose‐d_7_. The glucose signals include contributions from each label position and each anomer. Glucose‐d_7_ produces a broader composite spectrum compared to glucose‐d_2_, centred around approximately 3.6 ppm, along with additional resonances at approximately 5.2 and 4.6 ppm from the C1 deuterium atoms of the two anomers. The HDO, Glx and lactate+lipid signals show no obvious differences in linewidths and chemical shift positions between participants (or glucose isotopologues) and times after glucose ingestion. After approximately 100 min (see the decomposed spectra in Figure 1), the strongest enrichment can be seen for HDO, followed by glucose and Glx, while the lactate+lipid signal is less visually discernible. Figure 2 shows individual, temporally averaged CSI data covering the whole brain (top) for ingestion of glucose‐d_2_ and glucose‐d_7_, with representative temporally averaged single voxel spectral fits (middle) confirming the higher signal (more labelling) from glucose‐d_7_. The corresponding metabolite maps, interpolated to the resolution of the MPRAGE image, are shown in the bottom panels of Figure 2. At natural abundance, the average HDO signal‐to‐noise ratio (SNR) was 14 ± 1 in the CSI data and the linewidth averaged across subjects was 14 ± 1 Hz (values obtained from the 100 voxels with the largest HDO SNR for each subject were used for this calculation).

### 3.1 Improved Label Efficiency After Glucose‐d_2_ vs. Glucose‐d_7_ Ingestion

**FIGURE 3:**
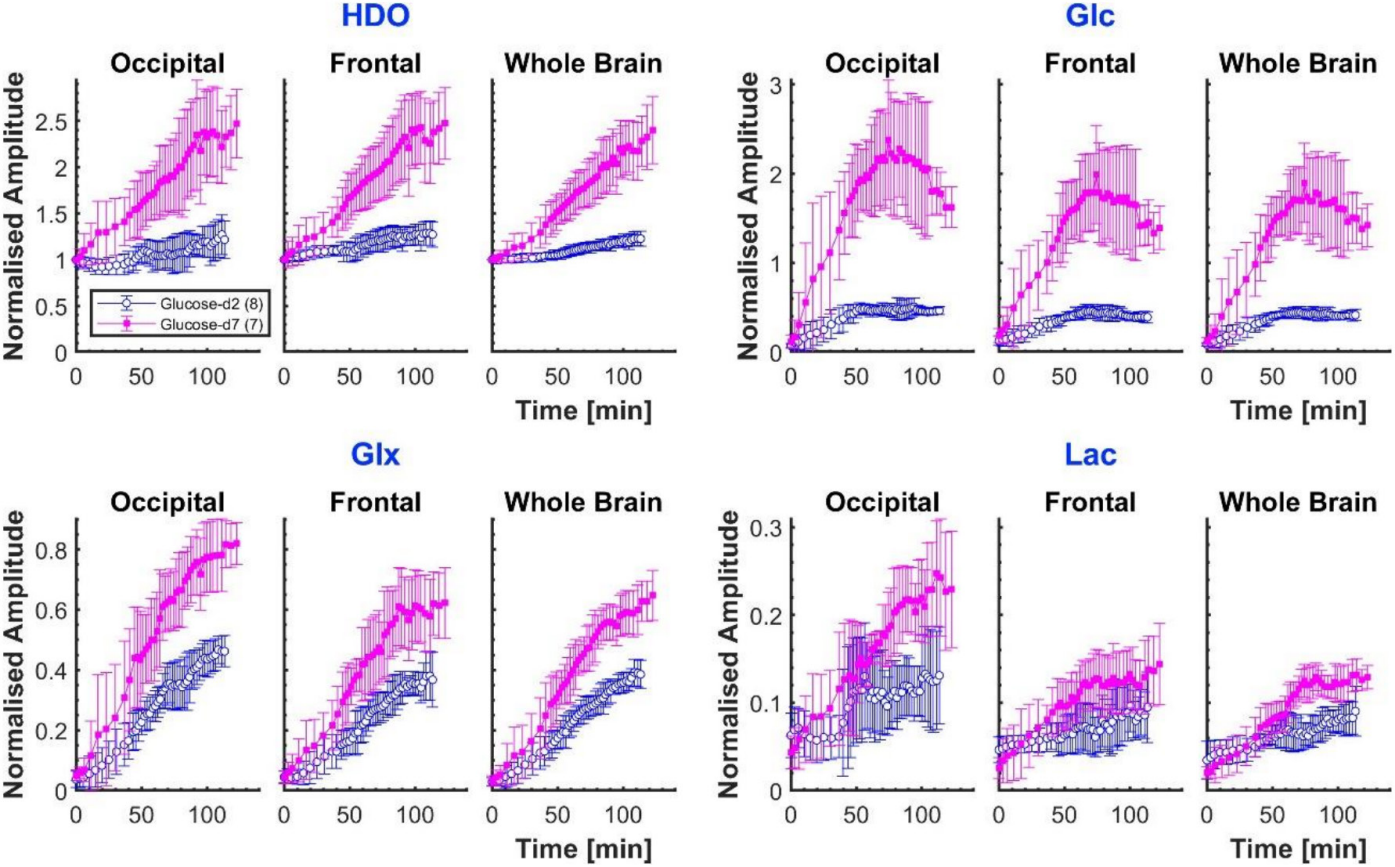
Time courses of normalised, average metabolite signal amplitudes: HDO, glucose, Glx and lactate. Amplitudes are averaged within a given ROI, for each participant, at each time point, followed by a moving average across the participant‐aggregated temporal data (with window size chosen to encompass a number of measurements equal to the number of participants in each cohort). The amplitudes are normalised to the natural abundance HDO signal amplitude measured at *t* = 0. Results from the occipital cortex, frontal cortex and whole brain are displayed for glucose‐d_2_ (blue) and glucose‐d_7_ (pink). Error bars are the moving standard deviations.

Participant‐averaged time courses of normalised metabolite signal amplitudes are displayed in Figure 3. The plots were produced by taking a moving average of the time‐ordered data from all participants in the glucose‐d_2_ or glucose‐d_7_ cohorts, with the window size chosen to encompass a number of measurements equal to the number of participants in each cohort. The temporal extent of the window consequently varies across the time course due to the varying times at which scans after ingestion of glucose were acquired in different subjects (the time window range was 13.0–17.7 min for glucose‐d_2_ and 12.6–20.5 min for glucose‐d_7_). The average and standard deviation of data in each window are plotted at the mean of the post‐ingestion time that the different measurements were acquired. These averaged amplitudes are normalised using the HDO signal at natural abundance, measured for each participant before ingestion of glucose.

The averaged time courses confirm that higher signals are produced from all metabolites after glucose‐d_7_ ingestion compared to glucose‐d_2_, but with marked gains for HDO and glucose, and less gain for Glx and lactate+lipids. The observed time courses differ substantially between metabolites and between glucose isotopologues. The HDO signal shows a near‐linear increase with time over the experimental time range of up to two hours after glucose ingestion (clearly steeper for glucose‐d_7_), while the glucose signal exhibits a maximum occurring between 70 and 90 min after glucose ingestion, and is higher in the occipital ROI than in the frontal and whole‐brain ROIs (see Tables S7 and S8 in Supplementary Material). The Glx and lactate+lipid time courses show reduced rates of increase with time at later time points, but do not peak within the two‐hour time frame of our observations. Time courses appear similar across ROIs although amplitudes show some interregional variation, again with higher values occurring in the occipital ROI (see Table S8 of the Supplementary Material). No apparent effect of visual stimulation could be discerned (see Figure S10 of the Supplementary Material) and, hence, data from experiments with and without visual stimulation were pooled for the analyses reported below.

**FIGURE 4:**
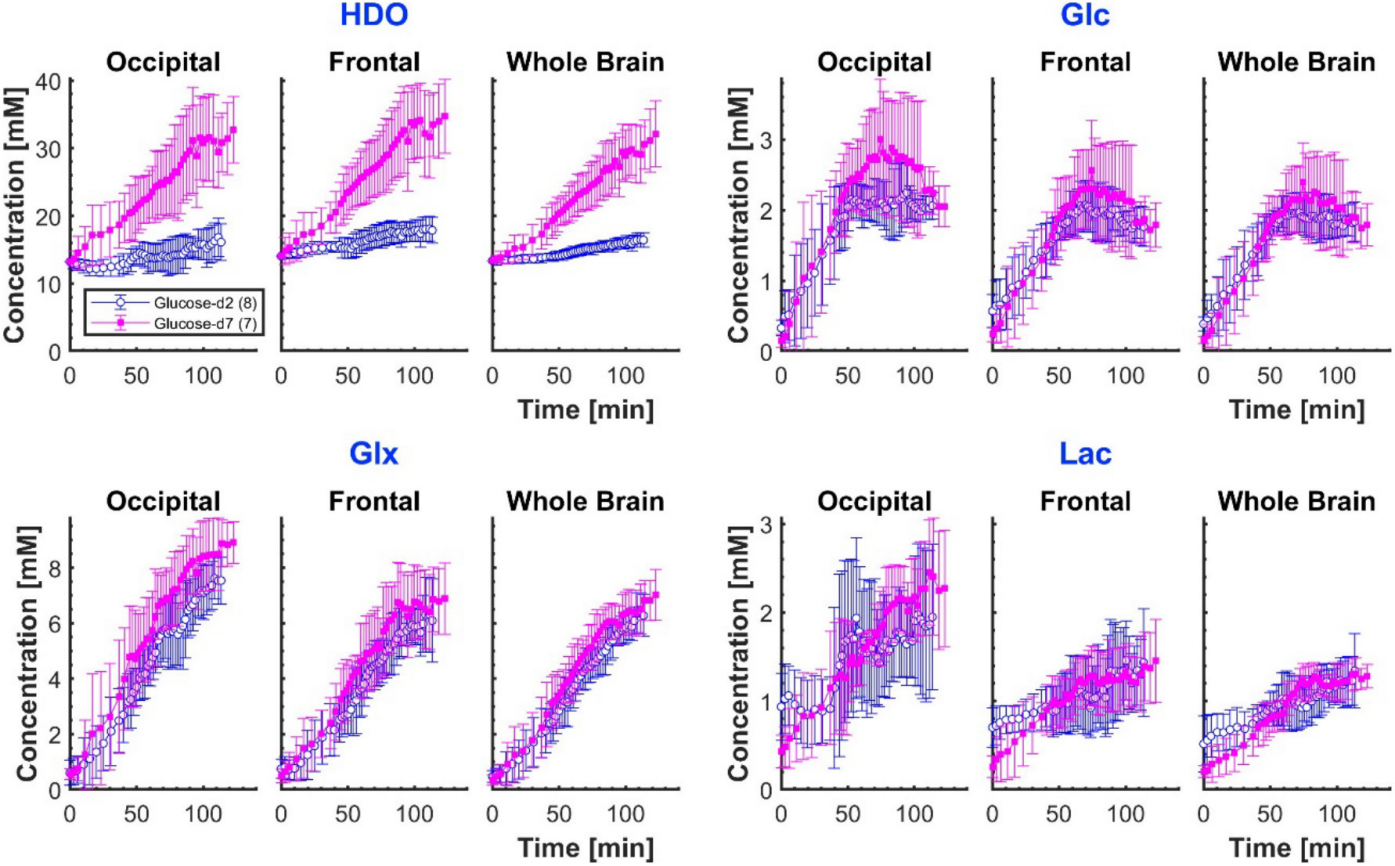
Time courses of average metabolite concentrations: HDO, glucose, Glx and lactate. Data treatment followed a similar process to that used for the time courses displayed in Figure 3, except that the metabolite amplitudes were converted to concentrations prior to averaging of the participant‐aggregated data.

The same data, converted to metabolite concentrations, are shown in Figure 4 (individual subject data are shown in Figure S6A and S6B of the Supplementary Material). These concentrations are corrected for label‐loss, so that the values are estimates of the concentrations of deuterated molecules that would have been produced had no label‐loss occurred, and the reported Lac values assume that the signal at 1.3 ppm is from lactate (with no lipid contamination). As expected, the averaged concentrations of HDO are clearly different between the glucose‐d_2_ and glucose‐d_7_ cohorts, but the glucose, Glx and lactate concentrations are similar. Lac and Glx concentrations were higher in the occipital versus frontal ROIs (for times between 80 and 100 min: Lac(d_2_) *p* = 0.03; Glx(d_2_) *p* = 0.07; Lac(d_7_) *p* = 10^−4^; Glx(d_7_) *p* = 0.04).

**FIGURE 5:**
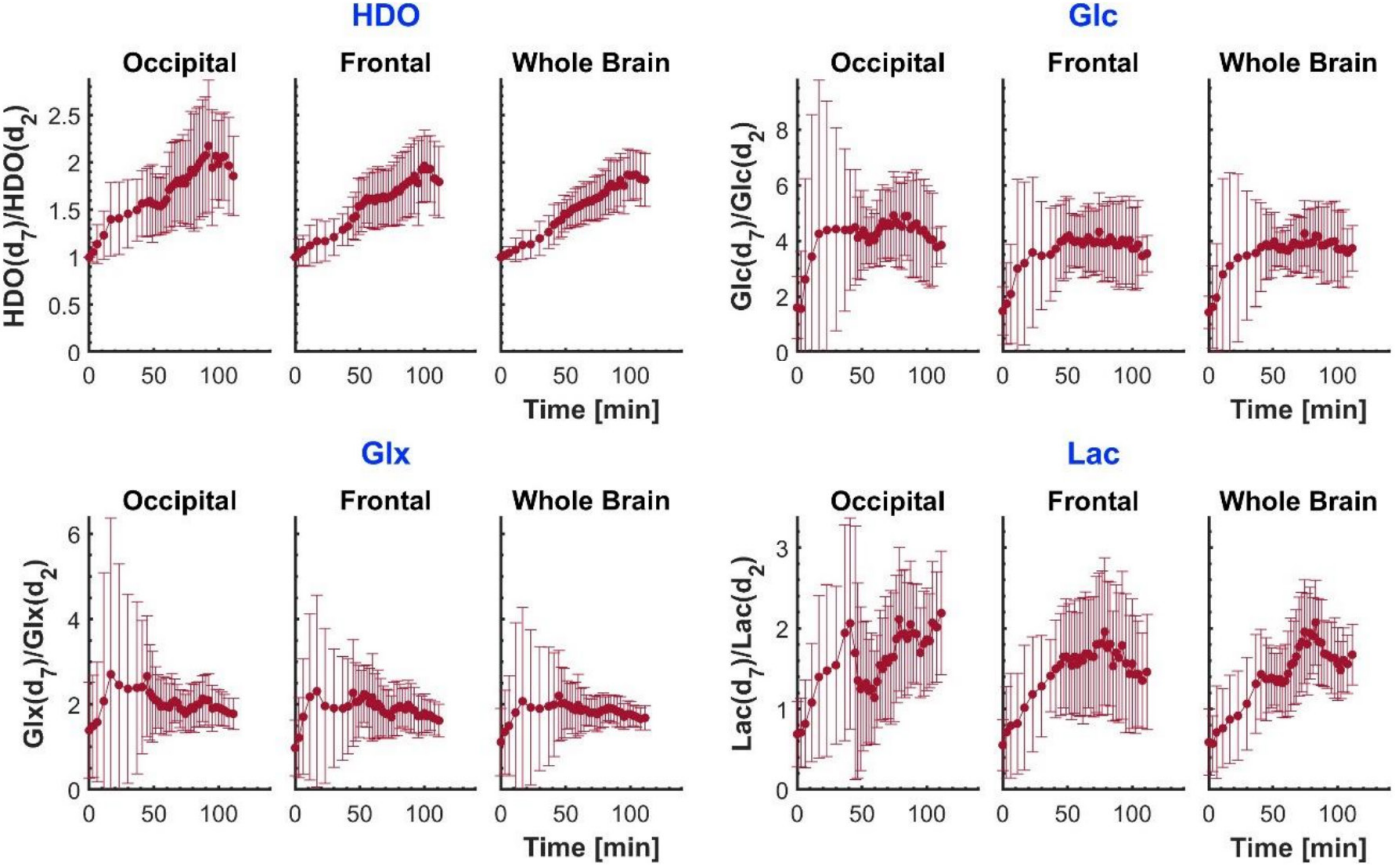
Amplitude ratios for metabolite, *m*, resulting from glucose‐d_7_ and glucose‐d_2_ ingestion, *m*(d_7_)/*m*(d_2_), where *m* (d_*i*_) is HDO, glucose, Glx, or lactate, produced by ingestion of glucose‐d_*i*_ (*i* = 2 or 7). In order to calculate the ratios, the glucose‐d_2_ metabolite amplitudes were interpolated to the same time points as the glucose‐d_7_ metabolite data. Results are averaged over participants.

Figure 5 shows the ratios of signals produced using the two different glucose isotopologues, i.e., *m*(d_7_)/*m*(d_2_), where *m* (d_*i*_) is the metabolite signal resulting from glucose‐d_*i*_ ingestion (*i* = 2, 7). The ratios show considerable variation over time but converge to approximately constant mean values (± std) for HDO, glucose, Glx and lactate+lipids, of 1.8 ± 0.3, 3.7 ± 0.8, 1.7 ± 0.3 and 1.6 ± 0.3, respectively, for whole‐brain data at times between 100 and 120 min. The HDO ratios reported here include the contribution of the NA deuterium in the water. Calculating instead the ratio of the signal change from natural abundance (ΔHDO) for the two isotopologues over the same time range gives a value of 8 ± 7, with the large error resulting from the large relative error in the ΔHDO values for glucose‐d_*2*_.

### 3.2 Dynamic Glucose Uptake and Metabolism

**FIGURE 6:**
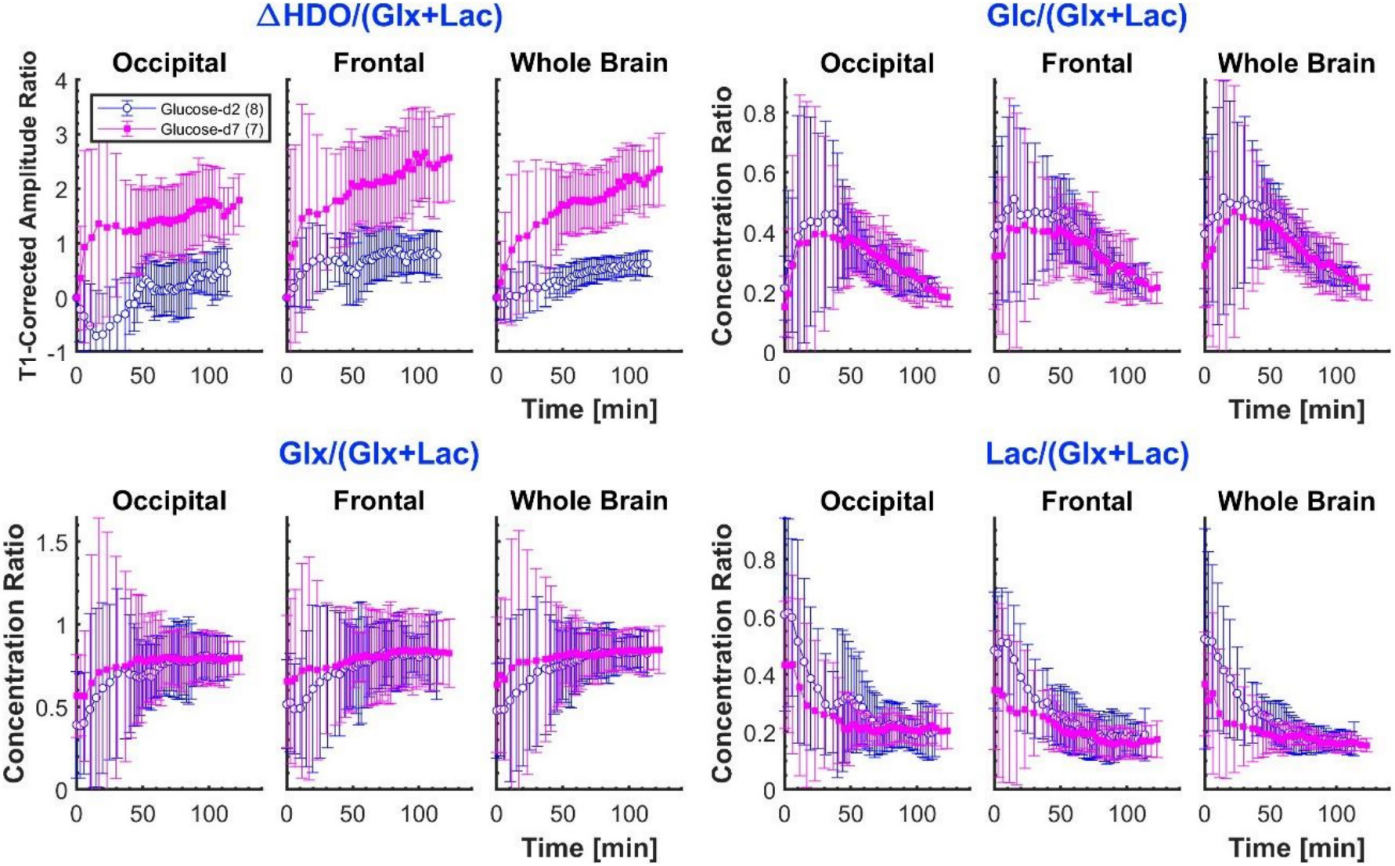
Ratio of each metabolite to the sum of Glx and lactate. For ΔHDO (top, left), the change in HDO above natural abundance, the ratio is of the T_1_‐corrected amplitudes. For Glc, Glx and Lac, the ratios are of concentrations.

To provide a clearer depiction of the relative metabolite signal amplitudes and concentrations that may better index healthy and altered glucose metabolism, the temporal variation of signal ratios after glucose‐d_2_ and glucose‐d_7_ ingestion is plotted in Figure 6. At early time points, there are large errors in these ratios because the low concentrations lead to low values of SNR. The T_1_‐corrected plots of ΔHDO/(Glx + Lac) for whole brain glucose‐d_7_ show values that steadily increase, reaching a quasi‐stable value of ~1.8 between 60 and 80 min, and then further increase to ~2.3 at 2 h. Average and individual participant values of these ratios at 95 min after glucose ingestion can be found in Table S9 of Supplementary Material. No significant effect between participants who were visually stimulated and those who were not was found for normalised amplitude or concentration following glucose‐d_2_ or glucose‐d_7_ ingestion; results are shown in Figure S10 of Supplementary Material. Plots of the concentration ratios Glx/(Glx + Lac) and Lac/(Glx + Lac) appear to reach a stable plateau earlier (after 50 min) of approximately 0.84 ± 0.02 and 0.16 ± 0.02, respectively, for glucose‐d_7_, while similar ratios are detected slightly later (from about 60 min) after glucose‐d_2_ ingestion. These ratios correspond to a concentration ratio of Lac/Glx = 0.21 ± 0.03 and 0.19 ± 0.03 for glucose‐d_2_ and glucose‐d_7_, respectively (Table S9). This is consistent with the data reported by Kaggie *et al* [48], which, when converted to a concentration ratio by scaling by the effective number of deuterium labels for Glx (0.6) and lactate (0.85) when using glucose‐d_2_, becomes 0.13 ± 0.06.

In the first 50–60 min, the Glx/(Glx + Lac) ratio in our data was much lower than the ‘steady‐state’ value found at later times, suggesting different dynamics of label enrichment between glycolytic and TCA pathways, which could be explained by sequential label turnover.

**FIGURE 7:**
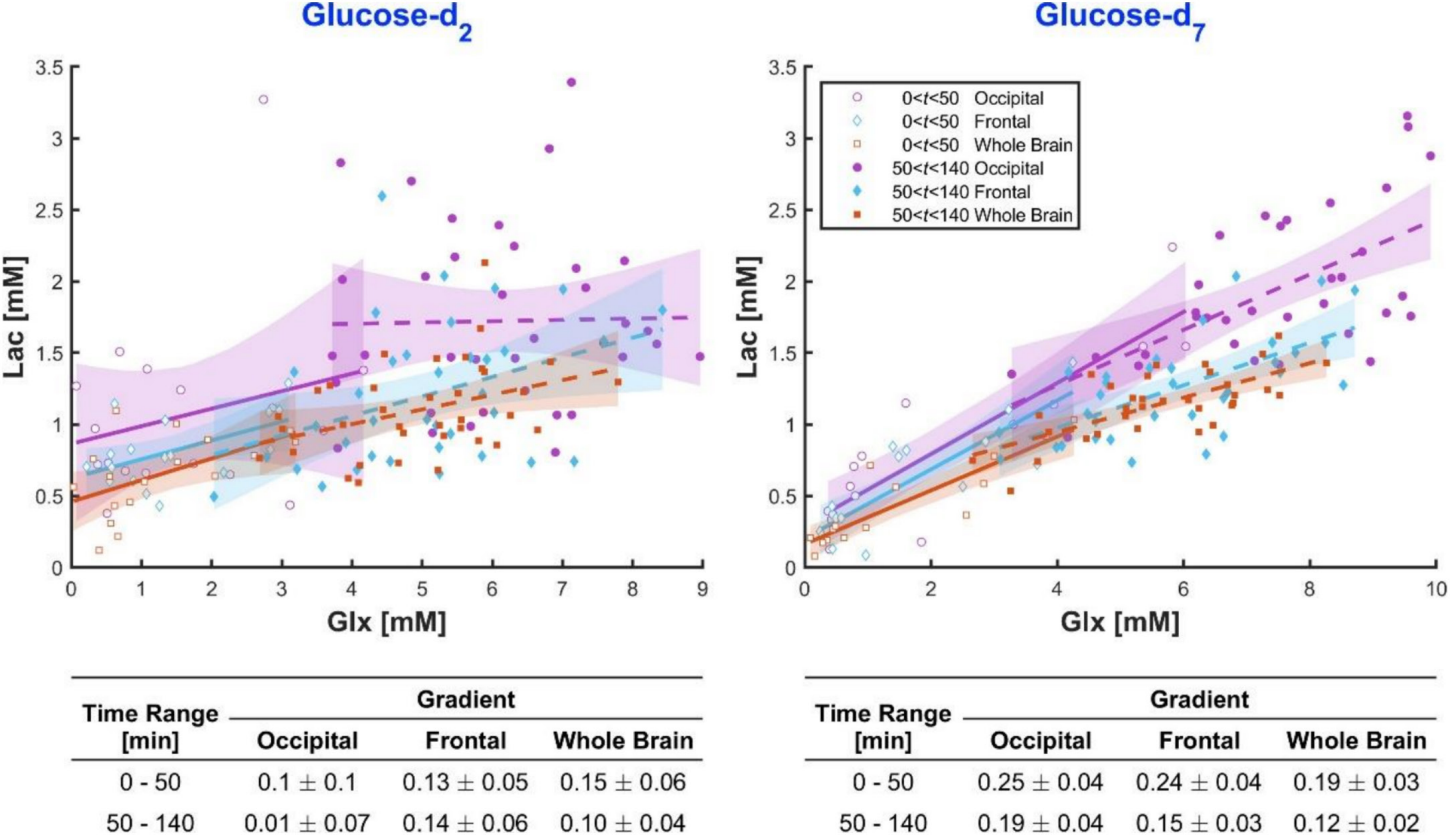
Lactate concentration against Glx concentration for all participants who ingested glucose‐d_2_ (left) and glucose‐d_7_ (right). All time points for CSI acquisitions are displayed but linear regressions are split into two ranges: 0–50 min and 50–140 min. Gradients are detailed for the two different time ranges and ROIs in the tables below each plot.

To yield more robust estimates of the relative glycolytic vs. TCA glucose consumption, we computed lactate vs. Glx regression plots (Figure 7) across all participants for all time points during the rise (0–50 min) and plateau phase (50–140 min), separately for both glucose isotopologues. Glucose‐d_7_ data confirm the higher glycolytic label ratio within the first 50 min (up to 0.25 for occipital and 0.24 for frontal), dropping to 0.19 (occipital) and 0.15 (frontal) from 50 min. Glucose‐d_2_ data show a similar non‐linear pattern for brain‐averaged ratios.

## 4 Discussion

Serial deuterium spectra and CSI data of the brain were acquired at 7 T from healthy participants before, and at 5 or 6 time points spanning a time range of 2 h after, ingestion of 0.75 g/kg of either glucose‐d_2_ or glucose‐d_7_. After glucose ingestion, deuterated water (HDO), glucose and Glx were detected in spectra from the brain for both glucose isotopologues. Lactate+lipid signals were also detected in most spectra but at a lower level, particularly for glucose‐d_2_, and were generally more challenging to detect, although average time courses from all participants revealed an unambiguous accumulation of a deuterated substance at the lactate+lipid resonance (see Figures 3 and 4). The example slice‐selective deuterium spectra, displayed in Figure 1, show the time evolution of signals from natural abundance to 100 min after glucose ingestion. These spectra, which have a higher SNR than the per‐voxel spectra from the CSI data, show signals from HDO (4.8 ppm), glucose (approximately 3.8 ppm) and Glx (2.4 ppm), with amplitudes being generally larger for the spectra of the participant who ingested glucose‐d_7_, especially for the HDO peak. Although possessing a very low SNR, a peak at 1.3 ppm is visible in some of these spectra and, again, generally appears to be larger in the glucose‐d_7_ spectra. Examples of spectral fits are also shown in Figure 1 for the spectra acquired at approximately 100 min following glucose‐d_2_ or glucose‐d_7_ ingestion. It can be seen from these plots that the spectral components, incorporating the anomeric decomposition of the two glucose isotopologues, fit well, with small residuals.

DMI using CSI is known to be affected by low SNR. Here, we explored the achievable spatio‐temporal resolution when combining 7 T with multi‐deuterated glucose. Despite a clear improvement in SNR following ingestion of glucose‐d_7_ compared to glucose‐d_2_, SNR was still low in the individual 11‐min data acquisitions, requiring spatial, temporal, or group averaging to yield robust metabolite estimates. An estimate of the data quality that would have been obtained by acquiring with a higher number of averages over a longer time can be produced by averaging several CSI acquisitions into a single data set. Figure 2 shows averages of all measurements acquired > 50 min after ingestion of glucose‐d_2_ and glucose‐d_7_. This combination of six CSI acquisitions would correspond to a single acquisition with 36 averages taking 125 min. This time could be reduced to 57 min by using acquisition‐weighted averaging [29]. It is worth noting that the combination of TR = 230 ms and FA = 62^o^ that we used here for the CSI acquisition is optimal for Lac and close to optimal for HDO in GM and WM. A larger FA at this TR would have delivered slightly higher SNR for Glx (x 1.07) and Glc (x 1.1) but would have reduced the SNR for HDO and Lac, and so FA = 62^o^ was chosen as a reasonable compromise.

Inspection of the averaged time course of glucose uptake and metabolite production in Figure 3 demonstrates a strong time evolution above natural abundance signals, following both glucose‐d_2_ and glucose‐d_7_ ingestion. ROI‐averaged metabolite amplitudes from individual participant data sets were often noisy (see Figure S6 of the Supplementary Material). This was more often the case for participants who ingested glucose‐d_2_, whose metabolite amplitudes tended to be lower but was also an issue for lactate+lipid for both glucose isotopologues. Averaging the data over all participants, however, as shown in Figures 3 and 4, delivered a clearer picture of the temporal accumulation of the deuterated metabolites. The curves for glucose clearly reach a global maximum within the timeframe of the experiments, and HDO, Glx and lactate+lipids, all unambiguously show increasing trends.

The ratios of measured metabolite signal amplitudes following glucose‐d_7_ and glucose‐d_2_ ingestion, as shown in Figure 5, can be predicted from the different numbers of deuterium labels arising from the two glucose isotopologues. Based on the fitting procedure used here, the ratio of signal amplitudes (Figure 5) for glucose‐d_7_ versus glucose‐d_2_ ingestion is expected to be 7/2 for glucose, and ignoring label loss would be 3/2 for both Glx and lactate. Label loss is important for Glx and lactate, but the loss factors have been assumed to be approximately the same for the two glucose isotopologues. The signal ratios for Glx and lactate are therefore unaffected by label loss and both remain at 3/2. The experimentally measured ratios are relatively stable for times greater than 50 min post‐ingestion, taking values at 100–120 min in the occipital, frontal and whole‐brain ROIs of 3.9 ± 0.4, 3.6 ± 0.6 and 3.7 ± 0.8 for glucose; 1.9 ± 0.5, 1.7 ± 0.4 and 1.7 ± 0.3 for Glx; 2.1 ± 0.7, 1.3 ± 0.4 and 1.6 ± 0.3 for lactate, which are generally slightly higher than the predicted values. The higher observed values could result from a larger average glucose uptake by the brain in the cohort who ingested glucose‐d_7_, as can be inferred by the higher peak concentrations shown in the glucose time courses of Figure 4.

Without label‐loss and ignoring any potential influx of HDO from metabolism in other tissues or other metabolic pathways, including deuterium atoms liberated during the TCA cycle and metabolism of Glx and lactate, there would be no increase in HDO signal above natural abundance from ingested glucose‐d_2_. With glucose‐d_7_, however, the four deuterium atoms bonded in the C2–C5 positions are all liberated to potentially become incorporated into water molecules during glycolysis [21, 22, 23]. If it is assumed that all deuterium atoms that are not incorporated into Glx or lactate due to label‐loss, contribute to an increase in HDO, then it is possible to estimate an upper value for the ratio of ΔHDO signals that should be produced by metabolism of the two glucose isotopologues. A calculation, detailed in the Supplementary Material, suggests that the average number of deuterium atoms lost from Glx and lactate that contribute to ∆ HDO is $∆N_{2}=0.72$ and $∆N_{7}=1.08$ per glucose molecule, for glucose‐d_2_ and glucose‐d_7_, respectively. An upper value for the ratio for ΔHDO is therefore $\left(4+∆N_{7}\right)/∆N_{2}=7.1$, where the numerical factor of four is due to the four deuterium atoms that are released during glycolysis from the C2–C5 positions. If the four deuterium atoms do not all become incorporated into water, forming HDO, then this ratio will be reduced. Also, contributions from the TCA cycle will increase both $∆N_{2}$ and $∆N_{7}$ which will also tend to reduce the above ratio. The small values of ΔHDO relative to the natural abundance HDO signal for glucose‐d_2_ lead to large variations in the calculation of the ratio of ΔHDO(d_7_)/ΔHDO(d_2_) but from our measurements we estimate a ratio of 8 ± 7. In Figure 5, however, we have shown the ratio HDO(d_7_)/HDO(d_2_). This ratio can be estimated by modifying the above calculation to include the concentration of HDO at natural abundance and an estimate of the total amount of glucose metabolised. This calculation, shown in the Supplementary Material, produces a HDO ratio of 2.3 ± 0.2, which is close to but slightly higher than our measured value of 1.8 ± 0.3. An estimate of total brain glucose load is required for this calculation: the results of this estimation are reported in the Supplementary Material in Figure S11, along with a description of the approach used.

The measured relative gains in signal amplitude for HDO, Glx and lactate+lipids following ingestion of glucose‐d_7_ versus glucose‐d_2_ translate directly into equivalent gains in SNR (i.e., SNR gains of 1.8 ± 0.3, 1.7 ± 0.3 and 1.6 ± 0.3 for whole‐brain data at times between 100 and 120 min). However, this is not the case for Glc since the glucose‐d_7_ spectrum contains multiple peaks (as can be seen in Figure 1), some of which overlap with the HDO signal. To evaluate the expected gain in SNR for the measurement of Glc signal amplitude, we therefore simulated FIDs from equivalent amounts of glucose‐d_7_ and glucose‐d_2_ and added appropriately weighted signal contributions from HDO, Glx and lactate+lipid, along with random noise to match the measured raw spectral SNR and linewidths in our CSI data. The fitting procedure was repeated 1000 times with different random noise added to the spectra, and the ratio of the mean fitted Glc amplitude to its standard deviation over repetitions was calculated. The results indicate that the SNR for glucose‐d_7_ is increased by a factor of 2.6 compared to glucose‐d_2_. As expected, this is lower than the 3.5‐fold increase in signal amplitude produced by the fitting procedure for equivalent glucose concentrations, but still represents a substantial gain in SNR.

As described above, a much stronger HDO signal enhancement was observed after glucose‐d_7_ ingestion, compared to glucose‐d_2_ ingestion, reaching a value of more than three times the natural abundance signal after 100 min. Mahar et al. [21] have suggested that the large increase in HDO signal from glucose‐d_7_ could be used as a metabolic biomarker that reflects glucose uptake and consumption, as is observed with [^18^F]fluorodeoxyglucose (FDG) PET. The increased HDO signal can potentially be monitored using ^2^H gradient‐echo imaging [22], making this a particularly simple measurement to implement. Since a larger glucose uptake should produce a larger amount of Glx and lactate, the HDO signal should also be related to the signal amplitudes of these metabolites. Previous in vivo ^2^H measurements on rat brain following intravenous injection of labelled glucose showed that a quasi‐stable region appears in a plot of ΔHDO/(Glx + Lac) against time from glucose infusion, which maintained a value of approximately 2.5 for a period of 15–20 min. In Figure 6, we have produced a similar plot (for glucose‐d_2_ as well as glucose‐d7, for comparison) and observed a delayed quasi‐stable ratio between approximately 50–80 min after glucose ingestion, but at a lower value of approximately 1.7. Mahar et al. [22] argued that the value of 2.5 is a consequence of label loss due to the action of alanine transaminase in conjunction with the four deuterium atoms liberated during the glycolysis pathway. They applied an unspecified correction to their data for other label losses occurring during glycolysis and the TCA cycle. Besides correcting for T_1_‐attenuation, we have not applied any other corrections to the data in Figure 6, which could explain the disagreement, but we would also consider the different protocols (ingestion vs. injection) and time scales as potential factors for the observed discrepancies with Mahar et al. beyond potential effects from anesthesia. Nevertheless, the strong and prolonged ΔHDO signal observed after glucose‐d_7_ ingestion may offer advantages over conventional DMI measurements of Glc, Glx and lactate for producing more highly resolved maps of combined glucose uptake and metabolism.

It is important to note that the benefits from performing DMI following ingestion of glucose‐d_7_ come at a fiscal cost as it is more expensive than glucose‐d_2_, at £130–£290 compared to £14–£19 per gram. Alternative methods of improving SNR include use of improved RF coil designs, increasing B_0_ [9], applying improved post‐processing denoising [49, 50] and improving acquisition sequences beyond standard CSI [50, 51]. In our experiments, we used a relatively large (26.4 cm id) dual‐tuned birdcage RF coil, and sensitivity could certainly be improved by using a tighter‐fitting receiver array, as has been used in other DMI work at fields of 7 T and above [12, 19]. It has been shown that as the applied B_0_ increases, the SNR of the ^2^H signal increases [9], proportionally to B_0_ with an exponent of 1.65. Therefore, to achieve increases of 1.8, 1.7 and 1.6 for HDO, Glx and Lac when using glucose‐d_7_ compared to glucose‐d_2_ at 7 T, a B_0_ field of around 10 T would be required, with a field of 12.5 T required to produce the calculated gain in SNR for Glc of 2.6. A subspace model with machine learning has been shown to improve SNR above standard CSI acquisitions of up to eight times, equivalent to our enhancement in our ΔHDO signal, and has also shown improvements compared to the Tucker decomposition used here [49]. The multi‐echo balanced steady‐state free precession (ME‐bSSFP) approach has been shown to improve SNR in MRSI acquisitions above standard CSI of up to 3–5 times [51]. However, this improvement was measured at 15.2 T, where ME‐bSSFP performance is enhanced relative to 7 T due to the increased spectral dispersion. Other sequences such as concentric ring trajectory (CRT) MRSI have been shown to provide improved spatial resolution and reduced scan times. In this case, SNR is reduced compared to Cartesian CSI acquisitions, but by leveraging low‐rank denoising techniques, this SNR loss is recoverable by the high dimensionality of the data [12]. The SNR in spectra can also be increased by increasing the voxel size. Each of these techniques has been shown to improve SNR of DMI acquisitions compared to standard CSI acquisitions, and cumulative improvements are possible if methods are combined.

The most obvious source of errors in this work was the fitting of the low‐SNR FIDs to determine the metabolite amplitudes, especially for the case of lactate+lipid. Additionally, in producing values for normalised signal amplitudes and metabolite concentrations, all metabolite amplitudes for a given participant were scaled by the measured HDO amplitudes at natural abundance, and errors in these amplitudes were consequently propagated to all subsequent time points. Errors in the natural abundance amplitudes could arise not just from the FID fitting but also potentially from imperfect registration of the sets of images from the scanning sessions before and after the glucose drink, which the participant consumed outside of the scanner. Despite these potential sources of error, the acquisition of a natural abundance CSI data set does provide benefits. Most obviously, it allows the voxel‐wise normalisation of metabolite amplitudes and, if tissue water concentrations are also estimated as prior knowledge, metabolite concentrations. In addition, in quantifying the metabolites via the natural abundance measurement, implicit account is taken of the actual spatial variation of the RF coil receive and transmit characteristics, unlike when, for example, normalising via measurements from the cerebellum or an external phantom [4]. In this study, there were specific issues which meant that we could not use the HDO concentration measured from the first CSI acquisition for normalisation. Since we aimed to gather data spanning times of up to 2 h after glucose ingestion while also limiting the time that subjects spent inside the scanner, we staggered the time of the first measurement made post‐ingestion across subjects. Hence, the first measurement in some cases was ~30 min after glucose ingestion, by which time the HDO concentration had changed significantly. This was a particular issue for glucose‐d_7_, since the HDO signal increases to 1.5 times the value at NA in the first 50 min after ingestion.

In converting signal amplitudes to concentrations for glucose‐d_7_, it was assumed that the effective number of deuterium labels for Glx and lactate was the same as those for glucose‐d_2_ (using values derived from data reported in de Graaf et al [45]) but scaled by a factor of 1.5, which reflects the ratio of the number of deuterium atoms, in the absence of label loss, that label the two pyruvate molecules produced by glycolysis. This assumption implies that the label loss for the deuterium atoms in the C1 position of glucose is similar to that in the C6 position. Ben‐Yoseph et al [52] studied the label loss from [1,6,6’‐^2^H_3_]glucose in rat glioma cells and found that there are two potential loss routes for the C1 label: a lesser mechanism via the pentose‐phosphate pathway and a greater mechanism caused by the action of phosphomannose isomerase on fructose‐6‐phosphate during glycolysis, together producing up to 52% loss for the C1 labels. We cannot claim that these mechanisms have not affected our data for glucose‐d_7_, but such additional losses would mean that the ratio of signal amplitudes for Glx and lactate, as shown in Figure 5, would tend to be less than 1.5, whereas the ratios are close to 1.5 or slightly larger. Funk et al [26] also measured label loss data for lactate and glutamate using glucose‐d_7_ in perfused rat hearts. Their data produce a similar effective label number for lactate (1.4, compared with 1.28) but a very different value for glutamate (0.33, compared with 0.9), and appears to be incompatible with our data since this would predict a much lower signal amplitude for Glx by a factor of 0.37 (assuming that glutamine has a similar label loss to glutamate). This apparent disagreement is possibly a result of comparing data from rat hearts [26] with data from rat brains [9].

In addition to label‐loss differences between the two glucose isotopologues, there is potentially also a difference in kinetic isotope effect (KIE). KIE is a measure of the reduced reaction rate caused by labelling molecules with deuterium and can also have an effect on pathway selection. KIEs have been measured for glucose‐d_2_ by de Graaf et al. [9] in rat brains and for glucose‐d_7_ by Funk et al. [26] in perfused rat hearts. The former researchers measured small, up to 4%, reductions in rates for lactate, glutamate and glutamine, while the latter measured no significant reduction for lactate but a larger reduction for glutamate. As with the label‐loss data from Funk et al. [26], it is unclear whether the KIE data is applicable to the human brain and, therefore, we do not currently know whether our measurements are affected by a KIE difference between the glucose isotopologues. However, since our relative concentrations (Figure 4) and signal amplitude ratios (Figure 5) are close to the predicted values, any differences in KIEs are probably not large but this does need further verification.

No significant difference for any metabolite, in any ROI, was found between participants who were subjected to a visual stimulus and those who were not. Since metabolite production rate and concentration changes due to visual stimulation have been reported in the literature [53, 54, 55], it is expected that such changes did occur during the experiments presented here but were of insufficient magnitude to be observed in the presence of measurement uncertainties and inter‐participant variability. The magnitude of any change in concentrations might have been less than maximal either because of non‐optimal timing of the visual stimulation or because the participants did not adequately maintain attention to the stimulus.

The increases in HDO and labelled Glx following ingestion of glucose‐d_2_ are as expected from many previous studies and consistent with aerobic glycolysis accounting for 10–12% of glucose consumption in the brain [56]. However, the observation of an increase in labelled lactate is less common, although it has been reported in previous DMI studies [7, 48] and is consistent with ^13^C MRSI measurements [37, 57]. Ruhm et al. [7] showed that an apparent lactate signal can arise from a non‐zero lipid component. Lipid contributions have approximately the same chemical shift as lactate at 1.3 ppm, and predominantly arise from subcutaneous adipose tissue. Therefore, it is expected that the selective spectra in Figure 1 will have contributions from lipids. However, the spectra shown in Figure 2 are from single voxels in the averaged CSI and have the same essential features as the slice‐selective spectra in Figure 11: clear HDO, glucose and Glx peaks, with low‐SNR peaks where lactate is expected. In these spectra, it is less likely that these peaks contain a large lipid contribution as the voxels are taken from a region with no overlap with subcutaneous fat. Example spectra from ~15 min and >50 min after glucose ingestion shown in Figure S13 provide clear evidence of increased lactate+lipid signal following glucose ingestion far away from subcutaneous fat. Also, the increased enhancement in lactate after ingesting glucose‐d_7_ compared to ingesting glucose‐d_2_ implies the lactate signal is not spurious, although voxels closer to the edge of the brain will have an increased chance of lipid contribution. While it is possible to remove the lipid contributions using spatial localisation by imaging (SLIM) in these edge voxels [42], we have not applied this method here.

## 5 Conclusion

We report a 7 T MRI protocol for the dynamic assessment of brain glucose uptake and metabolism after ingestion of glucose‐d_7_ compared to glucose‐d_2_ in healthy human volunteers. Detectable cerebral concentrations of glucose, unresolved glutamine/glutamate (Glx), and lactate were similar, but HDO was approximately 1.8 times larger with glucose‐d_7_. Estimates of glycolytic and oxidative metabolism were more precise when using glucose‐d_7_. Normative values show a strong temporal dependency of higher relative glycolytic activity within the first 50 min, with a subsequent equilibrium of a ratio of lactate to Glx production of up to 0.19 with some interregional variation. These findings suggest that the use of glucose‐d_7_ in conjunction with DMI can facilitate the non‐invasive in vivo assessment of metabolic reprogramming in the human brain, with a wide range of applications in experimental medicine and disease.

## Author Contributions

RB and DA conceptualised the project and provided supervision. ES, RD and DA were involved in writing ethics; all authors involved in project design. RD and DC were involved in data acquisition. RD and DC were involved in data analysis. DC, RD, ES, DA and RB were involved in original manuscript writing, and DC, RD and RB were involved in the review and revision stage.

## Conflicts of Interest

The authors declare no conflicts of interest.

## Supporting information


**Figure S1:** Study schematic. Participants consumed either glucose‐d_2_ or glucose‐d_7_ depending on the group to which they had been assigned at recruitment. The MR scanning occurred in two parts: the first 20‐min period was for baseline scans, and the second 90‐min period was the main scanning session in which deuterated metabolites were measured over time. Visual stimulation, if applied, occurred during the CSI scans only, as indicated by the blue blocks.
**Figure S2:** Masks of the occipital and frontal cortex along with the lateral ventricles shown in red‐yellow, blue and green respectively. Masks are shown overlaid on the MNI‐152 template with 1 mm isotropic voxels in sagittal, coronal and transverse orientations (left to right).
**Table S3:** Prior knowledge used in OXSA‐AMARES for the metabolites water (HDO), glucose (α and β anomers), Glx, and lactate. The table indicates lower bounds (LB), upper bounds (UB), initial value (IV) and the group (G). The latter indicates which metabolite components share a common or relative value.
**Table S4:** Net water percentages for ROIs, calculated from the percentage of constituent tissue‐type (determined by MNI segmentations and ROI atlas) and the percentage of water in each tissue type [2] (CSF 99%, GM 84% and WM 69%).
**Table S5:** Summary of T1 relaxation times of deuterated water (HDO) for tissues and ROIs. The values for tissue‐types CSF, GM and WM are taken from Cocking et al. [3]. The values for the ROIs (whole brain, occipital and frontal lobes) are calculated using the tissue percentages as shown in Table S4 and assuming fast‐exchange between all tissue compartments. T1 relaxation times for glucose, Glx and lactate of 67, 139 and 297 ms, respectively, are taken from De Feyter et al. [4], and assumed to be independent of tissue type, ROI and number of deuterium labels.
**Figure S6:** Metabolite concentration time courses for individual participants who ingested (A) glucose‐d_2_ (8 participants) and (B) glucose‐d_7_ (7 participants). Participants who received a visual stimulus are indicated by closed symbols. The curves are fits to the data from all participants, using the form $C\left(t\right)=A+B{\left(\text{kt}\right)}^{2}\text{exp}\left(-\text{kt}\right)$. The fitted parameters for the concentration data are displayed in Table S7.
**Table S7:** Fitted parameters {A,B,k} of the curve fits in Figure S6. r^2^ is the coefficient of determination for the nonlinear regression. F is a scaling factor to convert concentrations to normalised amplitudes, emphasising the fact that the data and regression lines of Figure S6 are essentially the same for concentrations and normalised amplitudes.
**Table S8:** Predictions from the fitted parameters {A,B,k} given in Table S7. C_max _= A + 4Be^−2^ is the predicted maximum concentration and occurs at T_max _= 2/k. C_100_ is the predicted concentration at 100 min.
**Table S9:** ΔHDO/(Glx+Lac) T_1_‐corrected amplitude ratios and Lac/Glx concentration ratios for all participants and ROIs at 95 min after glucose ingestion. The Lac/Glx concentration ratios can be converted to a ratio of T_1_‐corrected amplitudes by multiplying by the ratio of the number of deuterium labels: N^Lac^/N^Glx^ = 1.42 for glucose‐d_2_ and glucose‐d_7_.
**Figure S10:** Time courses of average metabolite concentrations, similar to those in Figure 4 of the main article, except that the data has been separated into four cohorts depending on the glucose isotopologue and whether the participants received a visual stimulation: glucose‐d_2_ with visual stimulation (green, closed symbols), without stimulation (blue, open symbols); glucose‐d_7_ with visual stimulation (pink, closed symbols), without stimulation (black, open symbols).
**Figure S11:** Estimation of glucose uptake. The participant‐averaged glucose curves above are created by summing the observed glucose concentration and half the sum of the Glx and lactate concentrations: Glc + (Glx + Lac)/2. The addition of Glx and lactate partially compensates for the decay of the glucose curve due to metabolism of glucose. Glucose‐d_2_ (blue, open symbols), glucose‐d_7_ (pink, closed symbols).
**Table S12:** MRSinMRS checklist.
**Figure S13:** Early (top left) and late (bottom left) spectra from the voxel highlighted in green in the middle images from a single subject. There is evident signal at 1.3 ppm in the late‐acquired average spectrum, which is not seen in the early data, and thus is unlikely to be due to lipids. Interpolated (top middle) and uninterpolated (right middle) lactate+lipid signal amplitude maps drawn from the same central slice in the late‐acquired data are also shown, the latter indicating relatively low signal at the periphery. The bottom right image shows a map of the HDO linewidth in Hz from the same data, indicating that there is some correspondence of regions with the lower linewidth and high lactate+lipid signal.

## Data Availability

The data that support the findings of this study are available from the corresponding author upon reasonable request.
