# Supplementary material for: Advancing Deuterium MRI to Track Human Cerebral Glucose Metabolism at 7 T: A Comparison of Glucose‐d2 and Glucose‐d7 Ingestion
Source: NMR Biomed. 2025 Nov 3;38(12):e70169. doi: 10.1002/nbm.70169 (PMC12583885; doi:10.1002/nbm.70169)
Supplement: Supplementary file 1 — Figure S1: Study schematic. Participants consumed either glucose‐d2 or glucose‐d7 depending on the group to which they had been assigned at recruitment. The MR scanning occurred in two parts: the first 20‐min period was for baseline scans, and the second 90‐min period was the main scanning session in which deuterated metabolites were measured over time. Visual stimulation, if applied, occurred during the CSI scans only, as indicated by the blue blocks. Figure S2: Masks of the occipital and frontal cortex along with the lateral ventricles shown in red‐yellow, blue and green respectively. Masks are shown overlaid on the MNI‐152 template with 1 mm isotropic voxels in sagittal, coronal and transverse orientations (left to right). Table S3: Prior knowledge used in OXSA‐AMARES for the metabolites water (HDO), glucose (α and β anomers), Glx, and lactate. The table indicates lower bounds (LB), upper bounds (UB), initial value (IV) and the group (G). The latter indicates which metabolite components share a common or relative value. Table S4: Net water percentages for ROIs, calculated from the percentage of constituent tissue‐type (determined by MNI segmentations and ROI atlas) and the percentage of water in each tissue type [2] (CSF 99%, GM 84% and WM 69%). Table S5: Summary of T1 relaxation times of deuterated water (HDO) for tissues and ROIs. The values for tissue‐types CSF, GM and WM are taken from Cocking et al. [3]. The values for the ROIs (whole brain, occipital and frontal lobes) are calculated using the tissue percentages as shown in Table S4 and assuming fast‐exchange between all tissue compartments. T1 relaxation times for glucose, Glx and lactate of 67, 139 and 297 ms, respectively, are taken from De Feyter et al. [4], and assumed to be independent of tissue type, ROI and number of deuterium labels. Figure S6: Metabolite concentration time courses for individual participants who ingested (A) glucose‐d2 (8 participants) and (B) glucose‐d7 (7 participants). Particip [file NBM-38-e70169-s001.docx]

**Supplementary Material**

**Advancing deuterium MRI to track human cerebral glucose metabolism at 7 T: A comparison of glucose-d_2_ and glucose-d_7_ ingestion**

**Daniel J Cocking^1,2^, Robin A Damion^1,3,4^, Elizabeth J Simpson^3,5^_,_ Dorothee P Auer^1,3,4^, and Richard Bowtell^1,2,3^**

1. Sir Peter Mansfield Imaging Centre, University of Nottingham, United Kingdom.
2. School of Physics and Astronomy, University of Nottingham, United Kingdom.
3. NIHR Nottingham Biomedical Research Centre/Nottingham Clinical Research Facilities, Queen's Medical Centre, United Kingdom.
4. Mental Health and Clinical Neurosciences, School of Medicine, University of Nottingham, United Kingdom.
5. School of Life Sciences, University of Nottingham, United Kingdom

**Visual Stimulation**

At the screening visit, participants were informed whether they would receive glucose-d_2_ or glucose-d_7_, and whether visual stimulation would be applied. Of those receiving glucose-d_2_ (*n*=8), 6 experienced a visual stimulus. Of those receiving glucose-d_7_ (*n*=7), 4 experienced a visual stimulus.

Visual stimulation was achieved via an 8 Hz flashing, black and white, radial checkerboard, similar to that used by Fernandes *et al.*^1^. The visual display was projected onto a screen that the participants could observe while lying in the scanner by wearing prism glasses. Most of the participants who experienced visual stimulation (three that ingested glucose-d_2_ and four that ingested glucose-d_7_) experienced a checkerboard flashing pattern that was active for 50 seconds followed by 10 seconds of a red cross on a grey background. However, two participants (both of whom ingested glucose-d_2_) experienced a checkerboard flashing pattern that was active for 30 seconds followed by 30 seconds of a red cross on a grey background. Participants who received no visual stimulation were asked to close their eyes. In all cases, the scanner room lights were turned off.

If the participant was to be visually stimulated, the display was activated during the CSI scans only and quiescent otherwise (see Figure S1).


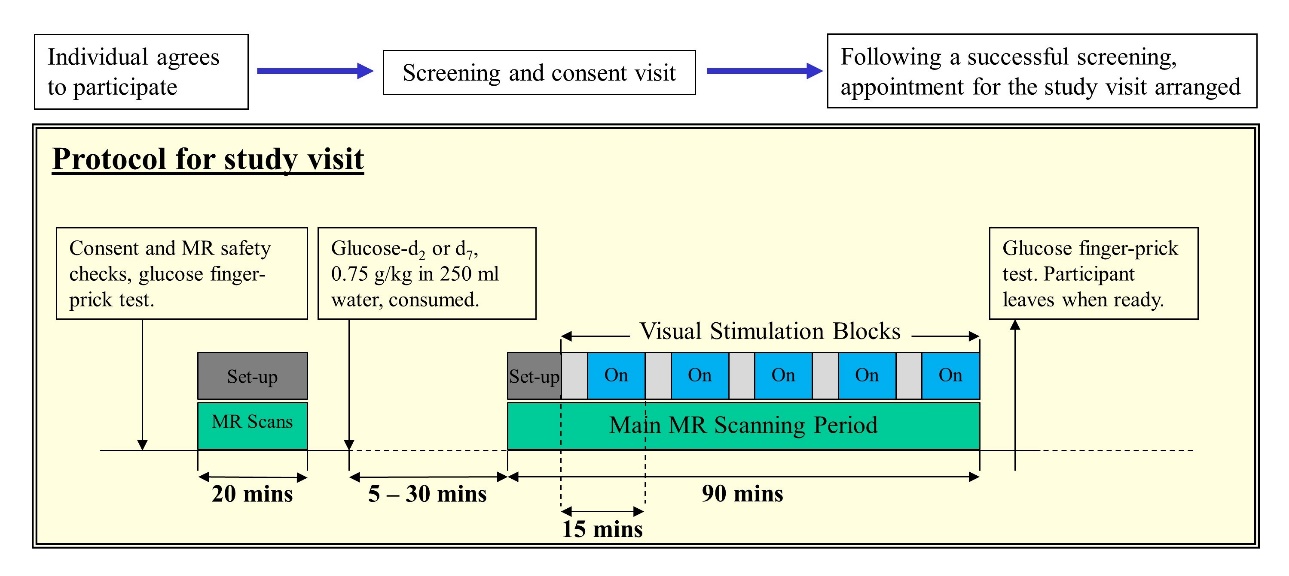


Figure S1. Study Schematic. Participants consumed either glucose-d_2_ or glucose-d_7_ depending on the group to which they had been assigned at recruitment. The MR scanning occurred in two parts: the first 20-minute period was for baseline scans, and the second 90-minute period was the main scanning session in which deuterated metabolites were measured over time. Visual stimulation, if applied, occurred during the CSI scans only, as indicated by the blue blocks.

**Masks of the regions of interest used**
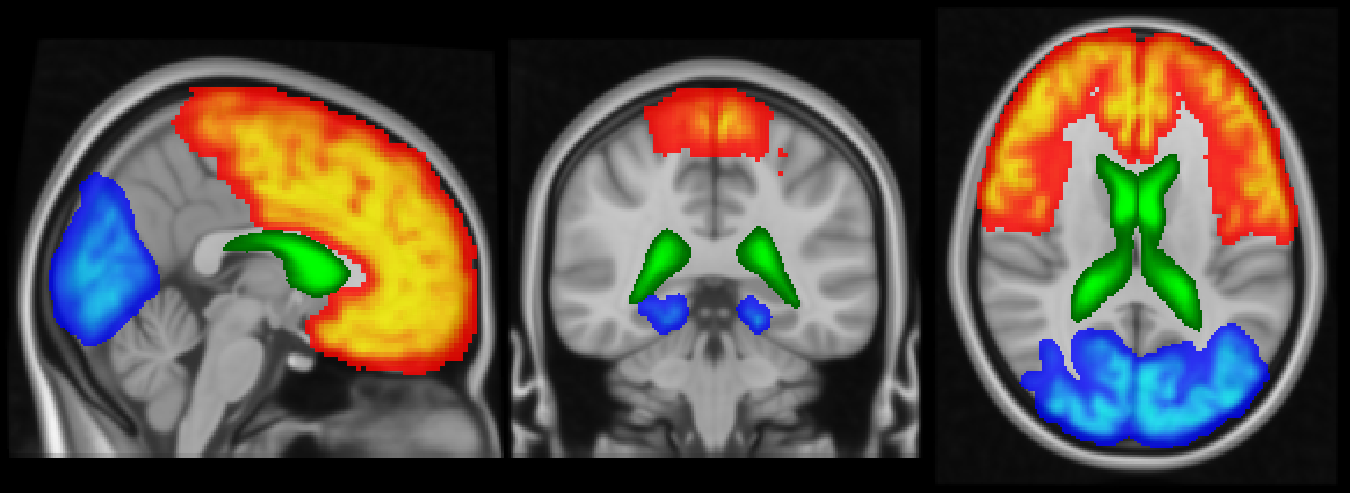


*Figure S2. Masks of the occipital and frontal cortex along with the lateral ventricles shown in red-yellow, blue and green respectively. Masks are shown overlaid on the MNI-152 template with 1 mm isotropic voxels in sagittal, coronal and transverse orientations (left to right).*

**Prior Knowledge of Fitting Parameters**

Table S3. Prior knowledge used in OXSA-AMARES for the metabolites water (HDO), glucose (α and β anomers), Glx, and lactate. The table indicates lower bounds (LB), upper bounds (UB), initial value (IV), and the group (G). The latter indicates which metabolite components share a common or relative value.


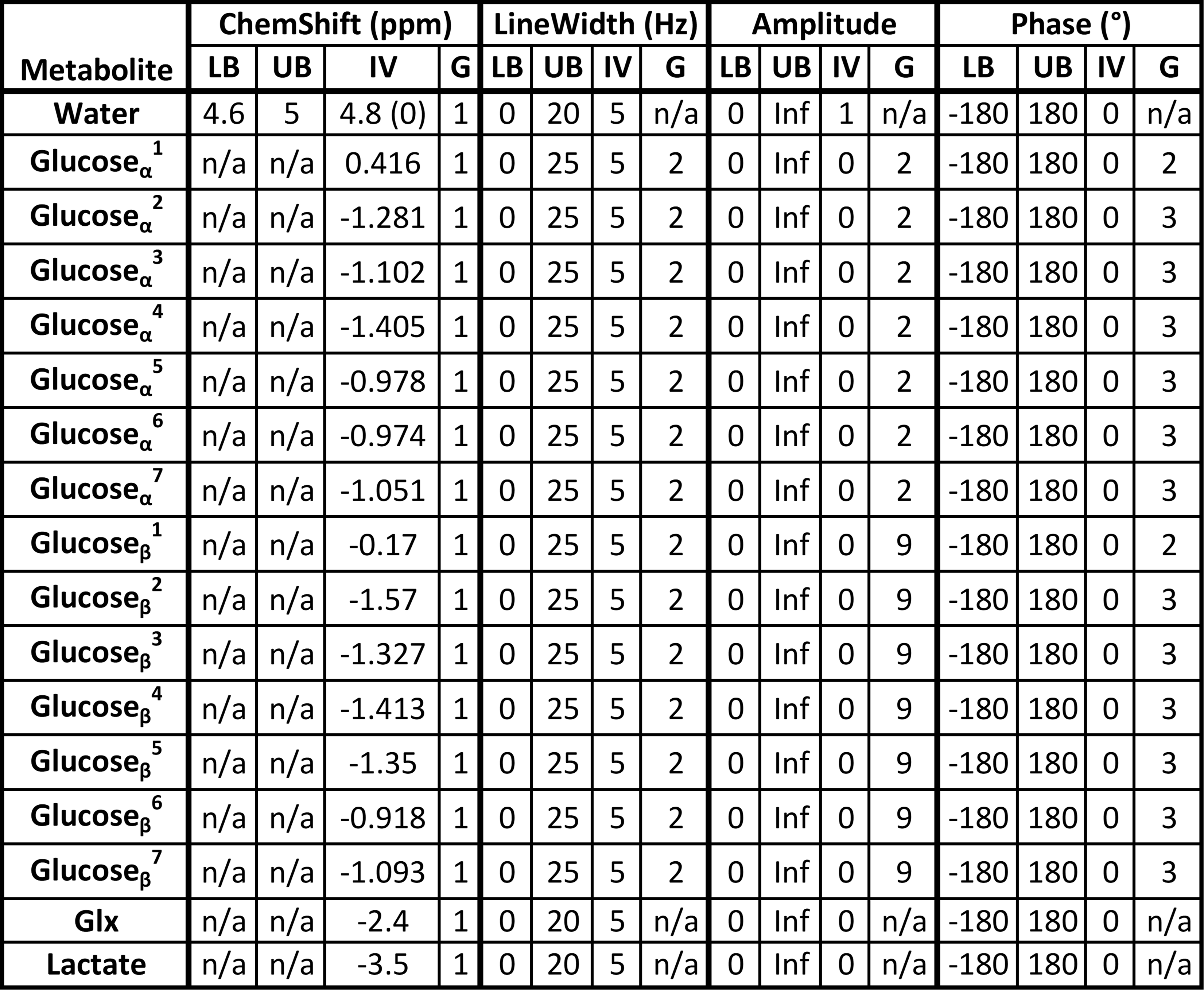


**Estimated Water Percentages and Longitudinal Relaxation Times for Tissues and ROIs**

Table S4. Net water percentages for ROIs, calculated from the percentage of constituent tissue-type (determined by MNI segmentations and ROI atlas) and the percentage of water in each tissue type^2^ (CSF 99%, GM 84%, and WM 69%).

| **ROI** | **Tissue Type %** | | | **Net Water %** |
| --- | --- | --- | --- | --- |
|  | **CSF** | **GM** | **WM** |  |
| Whole Brain | 23.7 | 40.3 | 36.0 | 82.2 |
| Occipital Lobe | 17.2 | 47.7 | 35.1 | 81.3 |
| Frontal Lobe | 33.8 | 47.3 | 18.9 | 86.2 |

Table S5. Summary of T_1_ relaxation times of deuterated water (HDO) for tissues and ROIs. The values for tissue-types CSF, GM, and WM are taken from Cocking et al.^3^. The values for the ROIs (whole brain, occipital and frontal lobes) are calculated using the tissue percentages as shown in Table S4 and assuming fast-exchange between all tissue compartments. T_1_ relaxation times for glucose, Glx and lactate of 67, 139, and 297 ms, respectively, are taken from De Feyter et al.^4^, and assumed to be independent of tissue type, ROI, and number of deuterium labels.

| **Tissue or ROI** | ***T*_1_ [ms]** |
| --- | --- |
| CSF | 510 |
| GM | 320 |
| WM | 290 |
| Whole Brain | 377 |
| Occipital Lobe | 329 |
| Frontal Lobe | 358 |

**Metabolite Concentration Time Courses of Participants**

1. **Glucose-d_2_**

**
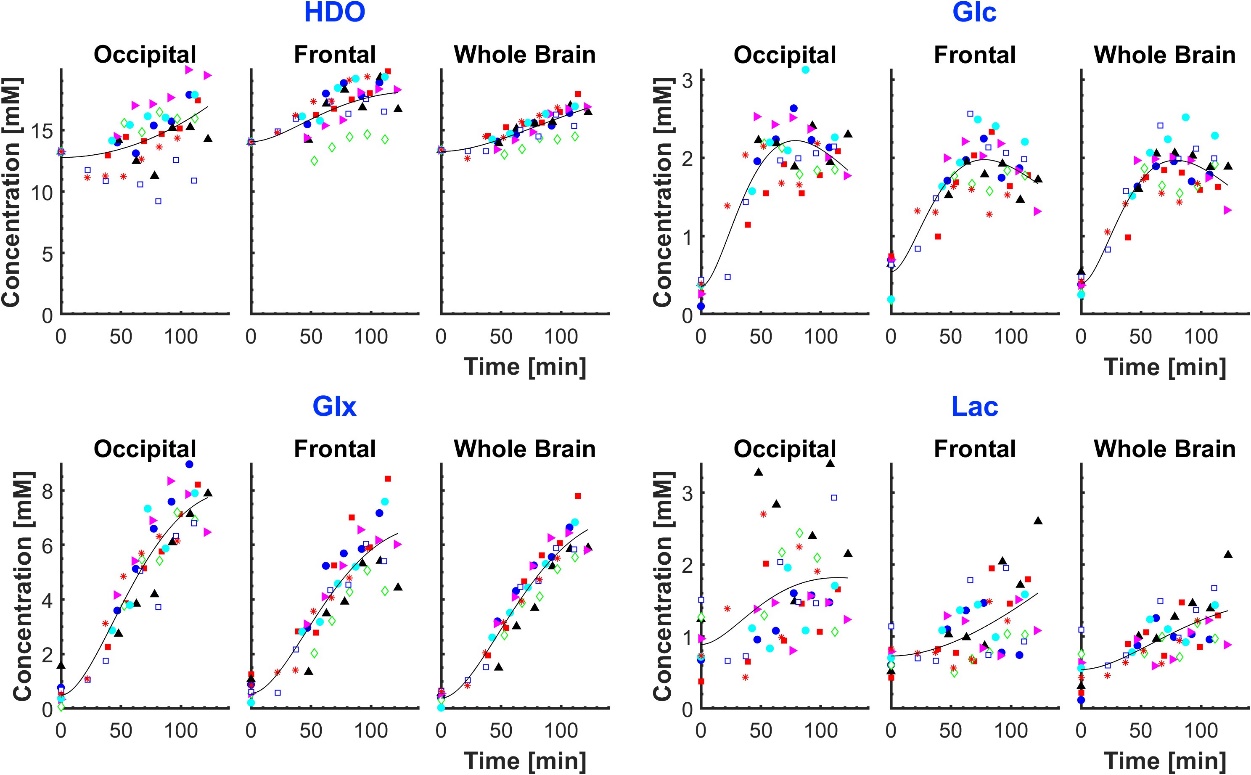
**

1. **Glucose-d_7_**


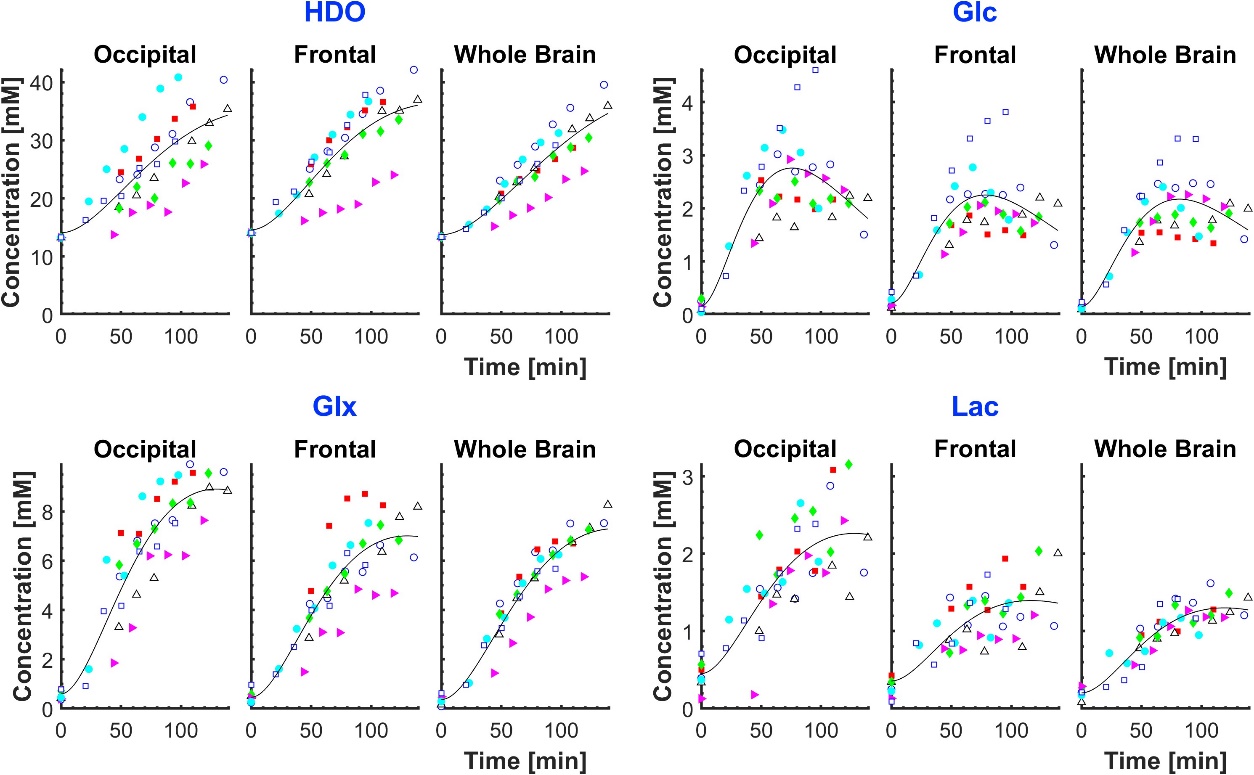


Figure S6. Metabolite concentration time courses for individual participants who ingested (A) glucose-d_2_ (8 participants) and (B) glucose-d_7_ (7 participants). Participants who received a visual stimulus are indicated by closed symbols. The curves are fits to the data from all participants, using the form $C\left( t \right)=A{+B\left( kt \right)}^{2}\text{exp}\left( -kt \right)$. The fitted parameters for the concentration data are displayed in Table S7.

**Nonlinear Regression for Metabolite Concentration Time Courses**

*Table S7. Fitted parameters* $\left\{ A,B,k \right\}$ *of the curve fits in Figure S6. r^2^ is the coefficient of determination for the nonlinear regression. F is a scaling factor to convert concentrations to normalised amplitudes, emphasising the fact that the data and regression lines of Figure S6 are essentially the same for concentrations and normalised amplitudes.*

| **Metabolite** | **ROI** | ***A***  **[mM]** | ***B***  **[mM]** | ***k***  **[min^-1^]** | ***r^2^*** | ***F* [mM]** |
| --- | --- | --- | --- | --- | --- | --- |
| **Glucose-d_2_** | | | | | | |
| HDO | Whole Brain | 13.3 ± 0.2 | 11 ± 6 | 0.007 ± 0.003 | 0.720 | 13.38 |
|  | Occipital | 12.8 ± 0.4 | 2.1x10^6^ ± 4x10^-11^ | 1.2x10^-5^ ± 1x10^-6^ | 0.280 | 13.25 |
|  | Frontal | 14.1 ± 0.4 | 7.6 ± 1.5 | 0.014 ± 0.004 | 0.487 | 14.05 |
| Glc | Whole Brain | 0.40 ± 0.08 | 2.9 ± 0.2 | 0.025 ± 0.001 | 0.849 | 4.42 |
|  | Occipital | 0.4 ± 0.1 | 3.4 ± 0.2 | 0.025 ± 0.001 | 0.803 | 4.42 |
|  | Frontal | 0.45 ± 0.09 | 2.6 ± 0.2 | 0.026 ± 0.002 | 0.767 | 4.50 |
| Glx | Whole Brain | 0.4 ± 0.2 | 12.2 ± 0.8 | 0.012 ± 0.001 | 0.940 | 16.27 |
|  | Occipital | 0.5 ± 0.3 | 13.8 ± 0.9 | 0.014 ± 0.001 | 0.900 | 16.30 |
|  | Frontal | 0.6 ± 0.3 | 11 ± 1 | 0.013 ± 0.002 | 0.844 | 16.58 |
| Lac | Whole Brain | 0.54 ± 0.09 | 1.8 ± 0.8 | 0.010 ± 0.005 | 0.490 | 14.84 |
|  | Occipital | 0.9 ± 0.2 | 1.7 ± 0.5 | 0.018 ± 0.006 | 0.214 | 15.12 |
|  | Frontal | 0.7 ± 0.1 | 10 ± 50 | 0.003 ± 0.009 | 0.315 | 14.86 |
| **Glucose-d_7_** | | | | | | |
| HDO | Whole Brain | 14 ± 1 | 46 ± 9 | 0.009 ± 0.002 | 0.827 | 13.38 |
|  | Occipital | 14 ± 2 | 40 ± 8 | 0.011 ± 0.003 | 0.638 | 13.25 |
|  | Frontal | 15 ± 1 | 40 ± 5 | 0.013 ± 0.002 | 0.727 | 14.05 |
| Glc | Whole Brain | 0.2 ± 0.1 | 3.7 ± 0.3 | 0.025 ± 0.002 | 0.733 | 1.26 |
|  | Occipital | 0.2 ± 0.2 | 4.8 ± 0.5 | 0.026 ± 0.002 | 0.699 | 1.26 |
|  | Frontal | 0.2 ± 0.2 | 3.8 ± 0.4 | 0.026 ± 0.002 | 0.666 | 1.29 |
| Glx | Whole Brain | 0.4 ± 0.2 | 12.8 ± 0.7 | 0.014 ± 0.001 | 0.924 | 10.85 |
|  | Occipital | 0.6 ± 0.4 | 15 ± 1 | 0.016 ± 0.001 | 0.860 | 10.86 |
|  | Frontal | 0.5 ± 0.4 | 12.1 ± 0.9 | 0.015 ± 0.002 | 0.815 | 11.06 |
| Lac | Whole Brain | 0.20 ± 0.06 | 2.0 ± 0.1 | 0.017 ± 0.001 | 0.851 | 9.89 |
|  | Occipital | 0.5 ± 0.1 | 3.3 ± 0.4 | 0.016 ± 0.002 | 0.686 | 9.91 |
|  | Frontal | 0.4 ± 0.1 | 1.9 ± 0.2 | 0.017 ± 0.003 | 0.585 | 10.08 |

*Table S8. Predictions from the fitted parameters* $\left\{ A,B,k \right\}$ *given in Table S7.* $C_{\max}=A+4Be^{-2}$ *is the predicted maximum concentration and occurs at* $T_{\max}=2/k$*.* $C_{100}$ *is the predicted concentration at 100 minutes.*

| **Metabolite** | **ROI** | ***T*_max_**  **[min]** | ***C*_max_**  **[mM]** | ***C*_100_**  **[mM]** |
| --- | --- | --- | --- | --- |
| **Glucose-d_2_** | | | | |
| HDO | Whole Brain | 270 ± 120 | 19 ± 3 | 16 ± 2 |
|  | Occipital | 1.7x10^5^ ± 2x10^4^ | 1.1x10^6^ ± 0.4 | 15.5 ± 0.8 |
|  | Frontal | 140 ± 40 | 18.2 ± 0.9 | 18 ± 1 |
| Glc | Whole Brain | 79 ± 3 | 2.0 ± 0.1 | 1.9 ± 0.1 |
|  | Occipital | 79 ± 4 | 2.2 ± 0.2 | 2.1 ± 0.2 |
|  | Frontal | 78 ± 5 | 2.0 ± 0.1 | 1.9 ± 0.1 |
| Glx | Whole Brain | 160 ± 10 | 7.0 ± 0.5 | 5.8 ± 0.5 |
|  | Occipital | 150 ± 10 | 8.0 ± 0.6 | 7.0 ± 0.7 |
|  | Frontal | 150 ± 20 | 6.7 ± 0.6 | 5.8 ± 0.7 |
| Lac | Whole Brain | 200 ± 90 | 1.5 ± 0.4 | 1.2 ± 0.4 |
|  | Occipital | 110 ± 40 | 1.8 ± 0.3 | 1.8 ± 0.3 |
|  | Frontal | 700 ± 2100 | 6 ± 27 | 1 ± 5 |
| **Glucose-d_7_** | | | | |
| HDO | Whole Brain | 220 ± 50 | 39 ± 5 | 29 ± 5 |
|  | Occipital | 180 ± 40 | 35 ± 5 | 30 ± 5 |
|  | Frontal | 160 ± 30 | 36 ± 3 | 33 ± 4 |
| Glc | Whole Brain | 82 ± 5 | 2.2 ± 0.2 | 2.1 ± 0.2 |
|  | Occipital | 76 ± 5 | 2.8 ± 0.3 | 2.5 ± 0.3 |
|  | Frontal | 78 ± 6 | 2.3 ± 0.3 | 2.1 ± 0.3 |
| Glx | Whole Brain | 150 ± 10 | 7.3 ± 0.4 | 6.5 ± 0.5 |
|  | Occipital | 130 ± 10 | 8.9 ± 0.7 | 8.4 ± 0.7 |
|  | Frontal | 130 ± 10 | 7.0 ± 0.6 | 6.6 ± 0.7 |
| Lac | Whole Brain | 119 ± 9 | 1.30 ± 0.09 | 1.27 ± 0.09 |
|  | Occipital | 130 ± 20 | 2.3 ± 0.2 | 2.2 ± 0.3 |
|  | Frontal | 120 ± 20 | 1.4 ± 0.2 | 1.4 ± 0.2 |

**ΔHDO/(Glx+Lac) and Lac/Glx**

*Table S9. ΔHDO/(Glx+Lac) T_1_-corrected amplitude ratios and Lac/Glx concentration ratios for all participants and ROIs at 95 minutes after glucose ingestion. The Lac/Glx concentration ratios can be converted to a ratio of T_1_-corrected amplitudes by multiplying by the ratio of the number of deuterium labels: N^Lac^/N^Glx^ = 1.42 for glucose-d_2_ and glucose-d_7_.*

|  | **ΔHDO/(Glx+Lac)** | | |  | **Lac/Glx** | | |
| --- | --- | --- | --- | --- | --- | --- | --- |
| **Participant** | **Whole Brain** | **Occipital** | **Frontal** |  | **Whole Brain** | **Occipital** | **Frontal** |
| **Glucose-d_2_** |  |  |  |  |  |  |  |
| P02 | 0.50 | 0.48 | 0.91 |  | 0.18 | 0.20 | 0.13 |
| P04 | 0.64 | 0.35 | 0.75 |  | 0.17 | 0.17 | 0.26 |
| P05 | 0.55 | 0.33 | 0.63 |  | 0.27 | 0.41 | 0.37 |
| P08 | 0.22 | 0.46 | 0.15 |  | 0.23 | 0.31 | 0.20 |
| P12 | 0.64 | 0.80 | 0.88 |  | 0.17 | 0.19 | 0.14 |
| P13 | 0.71 | 0.67 | 1.13 |  | 0.19 | 0.20 | 0.20 |
| P15 | 0.60 | -0.19 | 0.66 |  | 0.23 | 0.24 | 0.31 |
| P18 | 0.79 | 0.19 | 1.17 |  | 0.22 | 0.32 | 0.22 |
| Mean ± SD | 0.58 ± 0.16 | 0.38 ± 0.28 | 0.79 ± 0.30 |  | 0.21 ± 0.03 | 0.25 ± 0.08 | 0.23 ± 0.08 |
| **Glucose-d_7_** |  |  |  |  |  |  |  |
| P07 | 2.56 | 1.94 | 3.24 |  | 0.21 | 0.24 | 0.19 |
| P09 | 1.77 | 1.94 | 2.05 |  | 0.17 | 0.19 | 0.22 |
| P10 | 2.26 | 1.65 | 2.80 |  | 0.17 | 0.24 | 0.13 |
| P11 | 2.00 | 1.20 | 2.21 |  | 0.18 | 0.30 | 0.18 |
| P14 | 1.34 | 0.82 | 1.22 |  | 0.25 | 0.30 | 0.19 |
| P16 | 2.56 | 2.46 | 2.69 |  | 0.16 | 0.22 | 0.17 |
| P17 | 2.39 | 1.68 | 3.45 |  | 0.21 | 0.32 | 0.22 |
| Mean ± SD | 2.1 ± 0.4 | 1.7 ± 0.5 | 2.5 ± 0.7 |  | 0.19 ± 0.03 | 0.26 ± 0.04 | 0.19 ± 0.03 |

**
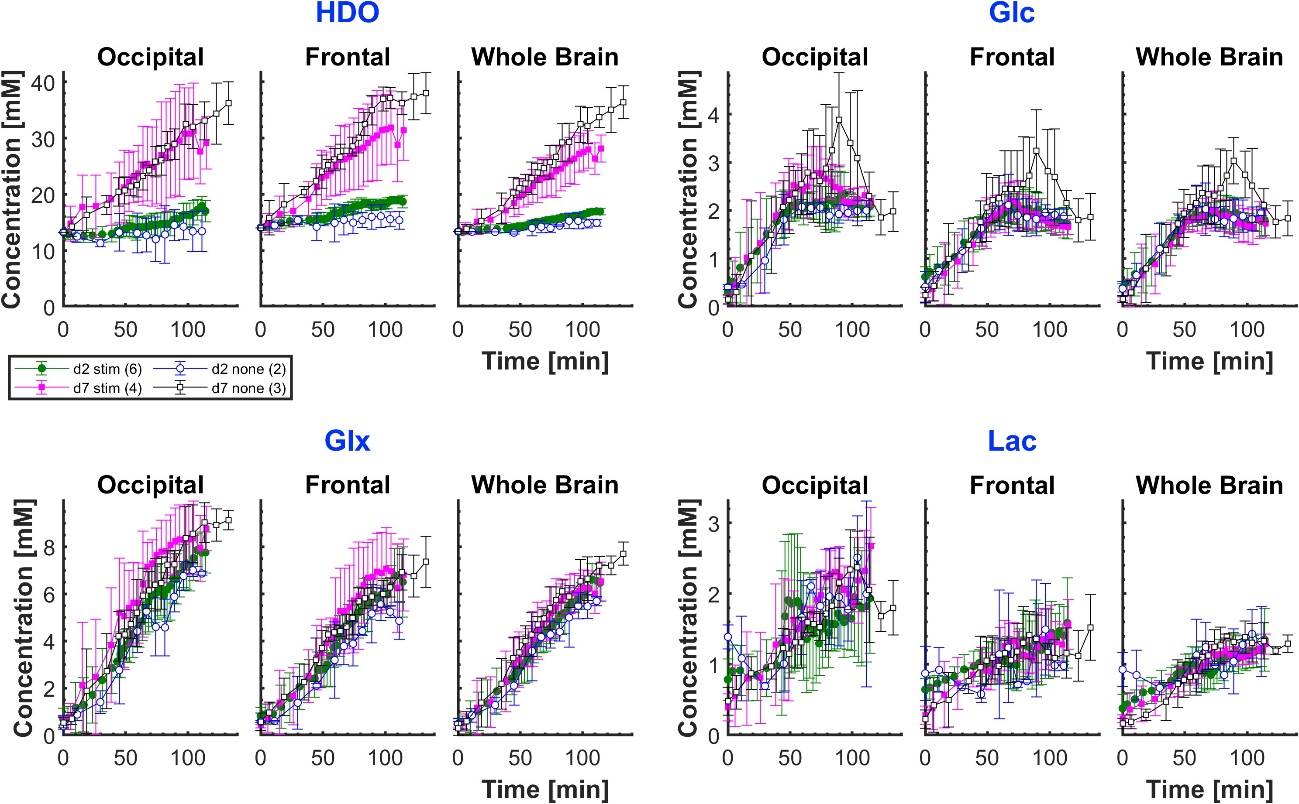
**

Figure S10. Time courses of average metabolite concentrations, similar to those in Figure 4 of the main article, except that the data has been separated into four cohorts depending on the glucose isotopologue and whether the participants received a visual stimulation: glucose-d_2_ with visual stimulation (green, closed symbols), without stimulation (blue, open symbols); glucose-d_7_ with visual stimulation (pink, closed symbols), without stimulation (black, open symbols).

**Estimation of ΔHDO(d_7_)/ΔHDO(d_2_) and HDO(d_7_)/HDO(d_2_)**

The average number of labels lost per glucose molecule is given by

| $\Delta N_{i}^{m}=2\left( \nu_{i}-N_{i}^{m} \right),$ | (1) |
| --- | --- |

where $m\in\left\{ G,L \right\}$ is the metabolite Glx or Lac, $i\in\left\{ 2,7 \right\}$ is the glucose isotopologue number, $\nu_{i}$ is the number of deuterium labels per metabolite molecule in the absence of label-loss, and $N_{i}^{m}$ is the effective number of deuterium labels per metabolite molecule with label-loss. The factor of two in (1) is because two pyruvate molecules are produced per glucose molecule.

Using the values (derived from data in de Graaf et al.^5^)

| $\nu_{2}=1, v_{7}=1.5,$ | (2) |
| --- | --- |
| $N_{2}^{G}=0.6, N_{2}^{L}=0.85, N_{7}^{G}=0.9, N_{7}^{L}=1.28,$ | (3) |

the average numbers of deuterium labels lost per glucose molecule are

| $\Delta N_{2}^{G}=0.8, \Delta N_{2}^{L}=0.3, \Delta N_{7}^{G}=1.2, \Delta N_{7}^{L}=0.44.$ | (4) |
| --- | --- |

To obtain an estimate of the contribution of these label-losses to HDO produced, per glucose molecule, the values for Glx and Lac need to be weighted by their concentration ratios. The ratio Glx/(Glx+Lac), shown in Figure 6 of the main article, is approximately 0.84 for both glucose isotopologues at later times (more than 50 minutes after glucose ingestion) and, therefore, we assume $f^{G}=0.84$ and $f^{L}=0.16$. Thus

| $\Delta N_{i}=f^{G}\Delta N_{i}^{G}+f^{L}\Delta N_{i}^{L},$ | (5) |
| --- | --- |

which produces the estimates

| $\Delta N_{2}=0.72, \Delta N_{7}=1.08.$ | (6) |
| --- | --- |

Combined with the four deuterium atoms that are liberated from glucose-d_7_ during glycolysis, the estimated ratio of ΔHDO is

| $\frac{\Delta\text{HDO}\left( \text{d}_{7} \right)}{\Delta\text{HDO}\left( \text{d}_{2} \right)}=\frac{\left( 4+\Delta N_{7} \right)}{\Delta N_{2}}=7.1.$ | (7) |
| --- | --- |

This value may be regarded as an upper bound because the four deuterium atoms liberated from glucose-d_7_ may not all become incorporated in a water molecule. Additionally, deuterium atoms liberated during the TCA cycle will contribute to HDO. Both considerations will tend to lower the ratio calculated above.

To calculate the ratio HDO(d_7_)/HDO(d_2_) , the above calculation needs to be modified to include the natural abundance of HDO and an estimate of the amount of metabolised glucose. That is,

| $\frac{\text{HDO}\left( \text{d}_{7} \right)}{\text{HDO}\left( \text{d}_{2} \right)}=\frac{{HDO}_{0}+\left( 4+\Delta N_{7} \right)\check{g}}{{HDO}_{0}+\Delta N_{2}\check{g}},$ | (8) |
| --- | --- |

where ${HDO}_{0}=13.4$ mM is the natural abundance of HDO for the whole brain ROI, and $\check{g}$ is the amount of metabolised glucose. In the calculation in the section below (Estimation of Total Brain Glucose Load) for the estimation of total glucose uptake, we estimate a value of 7 ± 1 mM. This value includes the observed glucose plus the metabolised glucose that produced Glx or lactate. From Figure 4 in the main article, it can be seen that in the long –term period, 100 – 120 minutes after ingestion, the observed glucose concentration is approximately 2 mM. Therefore, we estimate the metabolised glucose is 5 ± 1 mM. Therefore, from equation (8), the HDO ratio is 2.3 ± 0.2.

**Estimation of Total Brain Glucose Load**


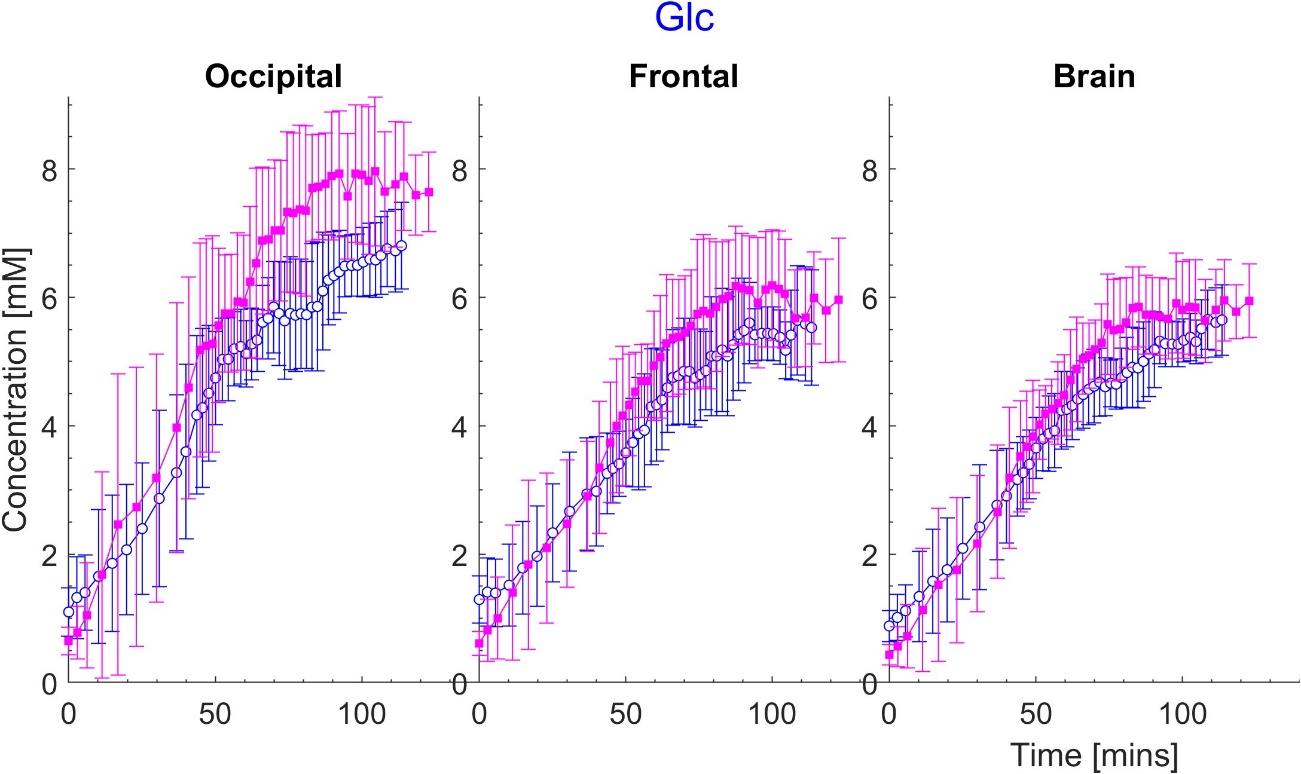


Figure S11. Estimation of glucose uptake. The participant-averaged glucose curves above are created by summing the observed glucose concentration and half the sum of the Glx and lactate concentrations: Glc+(Glx+Lac)/2. The addition of Glx and lactate partially compensates for the decay of the glucose curve due to metabolism of glucose. Glucose-d_2_ (blue, open symbols), glucose-d_7_ (pink, closed symbols).

Figure S11 shows that, on average, at least 6 mM of glucose entered the brain of the participants during this study. However, since a limited amount of data was acquired after the maximum of the estimated total glucose uptake curve, the value of 6 mM can only be regarded as an approximate lower estimate. However, there exists another way to obtain an estimate, as described below.

The simplest model of glucose uptake and metabolism is the first-order kinetic equation

| $\frac{dg}{dt}=\alpha\Gamma\left( t \right)-\beta g,$ | (9) |
| --- | --- |

where $g\left( t \right)$ is the observed glucose concentration, $\Gamma\left( t \right)$ is the glucose concentration arriving from the blood, $\alpha$ and $\beta$ are rate constants. The accumulation of observed glucose, $g$, does not directly reflect the influx, $\Gamma$, because of the loss term $-\beta g$, which accounts for glucose metabolism. If $\beta=0$, glucose would accumulate to a maximum value (assuming $\Gamma$ eventually decays to zero) and would reflect the influx and accumulation from the blood. In this scenario, let the glucose be denoted by $\tilde{g}$. Then

| $\frac{d\tilde{g}}{dt}=\alpha\Gamma\left( t \right),$ | (10) |
| --- | --- |

and then

| $\tilde{g}\left( \infty\right)=\int_{0}^{\infty} dt\frac{d\tilde{g}}{dt}=\int_{0}^{\infty} dt\alpha\Gamma\left( t \right),$ | (11) |
| --- | --- |

since $\tilde{g}\left( 0 \right)=0$. The asymptotic value $\tilde{g}\left( \infty\right)$ is therefore the total glucose uptake that we seek.

Substituting equation (9) into (11), we have

| $\tilde{g}\left( \infty\right)=\int_{0}^{\infty} dt\left( \frac{dg}{dt}+\beta g \right)=g\left( \infty\right)-g\left( 0 \right)+\beta\int_{0}^{\infty} dtg.$ | (12) |
| --- | --- |

Since, however, we assume that the observed glucose has boundary conditions $g\left( \infty\right)=g\left( 0 \right)=0,$ we simply have

| $\tilde{g}\left( \infty\right)=\beta\int_{0}^{\infty} dtg.$ | (13) |
| --- | --- |

Figure S6 shows fitted curves to concentrations in which the fitting function was assumed to be of the form

| $g\left( t \right)=A+B\left( kt \right)^{2}e^{-kt}.$ | (14) |
| --- | --- |

The constant, $A$, is not essential and merely captures a finite value at $t=0$ due to noise. Assuming that this constant can be ignored, thus setting $A=0,$ the derivative of (14) is

| $\frac{dg}{dt}=2Bk^{2}te^{-kt}-kg.$ | (15) |
| --- | --- |

This differential equation is of the form of equation (8) and identifies $k=\beta$. Therefore, we can perform the integral in (13) as

| $\tilde{g}\left( \infty\right)=kB\int_{0}^{\infty} dt\left( kt \right)^{2}e^{-kt}=B\int_{0}^{\infty} duu^{2}e^{-u}=2B.$ | (16) |
| --- | --- |

From the fitted values in Table S7 for glucose in the whole brain ROI, we obtain the estimates for the total brain glucose load of 5.8 ± 0.4 mM for glucose-d_2_ and 7.4 ± 0.6 mM for glucose-d_7_, which closely match the concentration estimates from Figure S11.

***Percentage of consumed dose***

From Table 1 in the main article, the average mass of participants was 63 ± 9 kg and 67 ± 15 kg for those who ingested glucose-d_2_ and glucose-d_7_ respectively. At a glucose dose of 0.75 g/kg, these equate to 47 ± 7 g and 50 ± 11 g of glucose-d_2_ and glucose-d_7_. The molar masses are respectively approximately 182 g and 187 g, and therefore the average amount of ingested glucose was 260 ± 40 mmol and 270 ± 60 mmol. Assuming an average human brain volume of 1.2 L, the concentration estimates in the preceding paragraph become 7.0 ± 0.5 mmol for glucose-d_2_ and 8.9 ± 0.7 mmol for glucose-d_7_ which are 2.7% ± 0.5% and 3.3% ± 0.8% of the ingested amounts.

**Minimum reporting standards in magnetic resonance spectroscopy (MRSinMRS)**

Minimum reporting standards checklist according to consensus in Lin et al^6^

*Table S12. MRSinMRS checklist.*

| 1. Hardware |  |
| --- | --- |
| a. Field strength [T] | 7T |
| b. Manufacturer | Philips Healthcare |
| c. Model (software version if available) | Acheiva |
| d. RF coils: nuclei (transmit/ receive), number of channels, type, body part | Dual-tuned ^2^H/^1^H Single-Channel Birdcage head coil, Rapid Biomedical |
| e. Additional hardware | 4-kW amplifier (CPC, New York USA) that operated at lower frequencies (10 – 130 MHz) spanning the 2H resonance frequency. |
| 2. Acquisition |  |
| a. Pulse sequence | CSI |
| b. Volume of Interest (VOI) locations | Whole Brain |
| c. Nominal VOI size [cm^3^, mm^3^] | 180 x 180 x 120 mm^3^ |
| d. Repetition Time (TR), Echo Time (TE) [ms, s] | TR = 230 ms, TE = 2.4 ms |
| e. Total number of Excitations or acquisitions per spectrum in time series for kinetic studies   1. Number of Averaged spectra (NA) per time-point 2. Averaging method (e.g. block-wise or moving average) 3. Total number of spectra (acquired / in time-series) | 6 acquisition-weighted averages  5/6 measurements acquired per scan session |
| f. Additional sequence parameters (spectral width in Hz, number of spectral points, frequency offsets)  If STEAM:, Mixing Time (TM)  If MRSI: 2D or 3D, FOV in all directions, matrix size, acceleration factors, sampling method | Bandwidth = 1200 Hz, Flip-angle = 62°, samples = 256, scan duration = 670s |
| g. Water Suppression Method | N/A |
| h. Shimming Method, reference peak, and thresholds for “acceptance of shim” chosen | Automated shimming up to 2^nd^ order was performed over the imaging volume using a pencil beam approach to map the field inhomogeneity along projections. (R Gruetter, 1993). |
| i. Triggering or motion correction method (respiratory, peripheral, cardiac triggering, incl. device used and delays) | N/A |
| 3. Data analysis methods and outputs |  |
| a. Analysis software | OXSA-AMARES |
| b. Processing steps deviating from quoted reference or product | Adapted to include ^2^H nucleus, and include chemical shifts reference to water and grouped. |
| c. Output measure (e.g. absolute concentration, institutional units, ratio)Processing steps deviating from quoted reference or product | Normalised Values, Concentrations |
| d. Quantification references and assumptions, fitting model assumptions | Reference to assumed water concentration, accounting for label-loss, number of ^2^H labels and T_1_ correction. |
| 4. Data Quality |  |
| a. Reported variables (SNR, Linewidth (with reference peaks)) | Natural abundance SNR = 14 ± 4, HDO Linewidths = 14 ± 1 Hz. |
| b. Data exclusion criteria | No exclusion criteria |
| c. Quality measures of postprocessing Model fitting (e.g. CRLB, goodness of fit, SD of residual) | No quality measure of postprocessing reported. |
| d. Sample Spectrum | Figures 1 and 2 |

**Figures indicating lactate accumulation in a single participant**

*
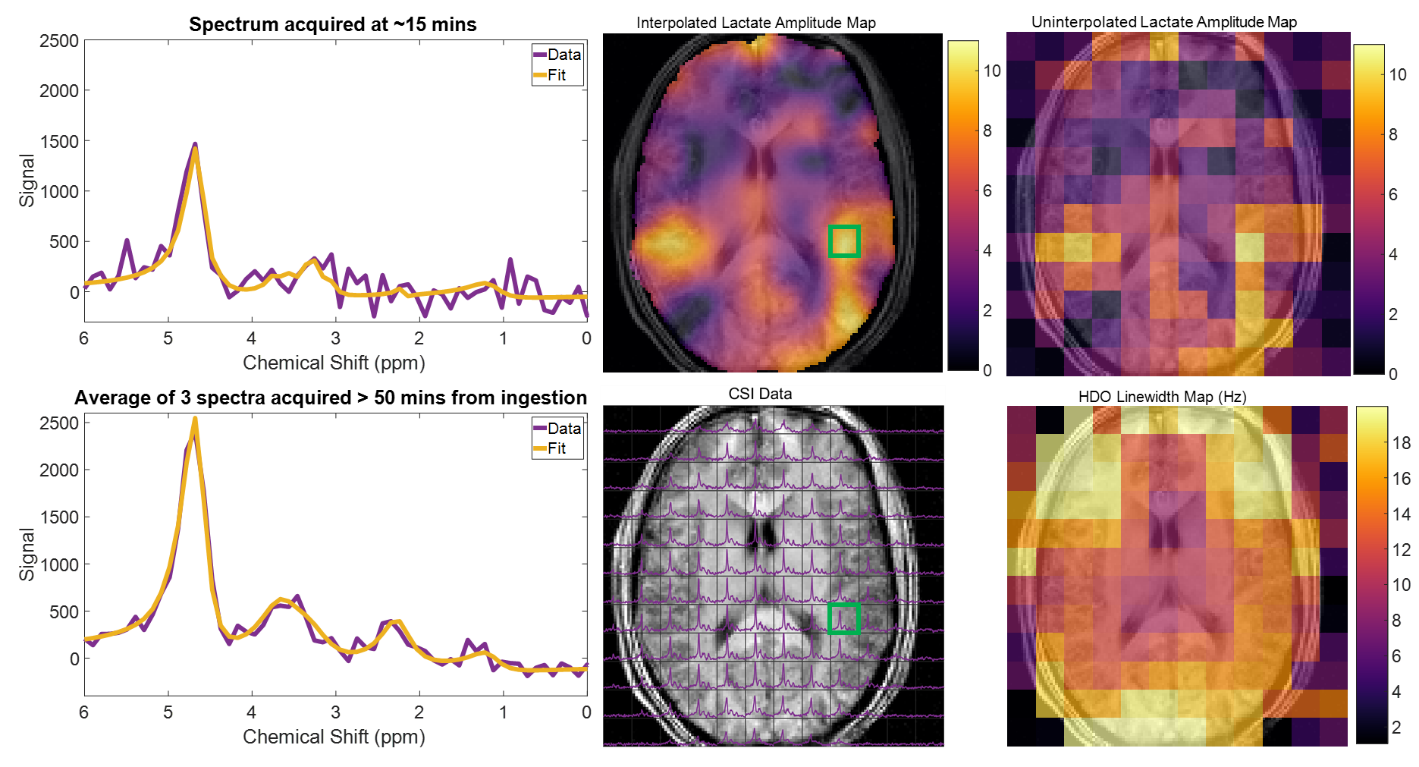
Figure S13. Early (top left) and late (bottom left) spectra from the voxel highlighted in green in the middle images from a single subject. There is evident signal at 1.3 ppm in the late-acquired average spectrum, which is not seen in the early data, and thus is unlikely to be due to lipids. Interpolated (top middle) and uninterpolated (right middle) lactate+lipid signal amplitude maps drawn from the same central slice in the late-acquired data are also shown, the latter indicating relatively low signal at the periphery. The bottom right image shows a map of the HDO linewidth in Hz from the same data, indicating that there is some correspondence of regions with the lower linewidth and high lactate+lipid signal*

**References**

1. Fernandes CC, Lanz, B, Chen C, Morris PG. Measurement of brain lactate during visual stimulation using a long TE semi-LASER sequence at 7T. NMR in Biomedicine. 2020; 33: e4223. doi.org/10.1002/nbm.4223
2. Oros-Peusquens AM, Loução R, Abbas Z, Gras V, Zimmermann M, Shah NJ. A Single-Scan, Rapid Whole-Brain Protocol for Quantitative Water Content Mapping With Neurobiological Implications. *Front Neurol*; 2019; 10. DOI: 10.3389/fneur.2019.01333.
3. Cocking D, Damion RA, Franks H, Jaconelli M, Wilkinson D, Brook M, Auer DP, Bowtell R. Deuterium brain imaging at 7T during D2O dosing. *Magn Reson Med* 2023; 89: 1514–1521. doi.org/10.1002/mrm.29539
4. De Feyter HM, Behar KL, Corbin ZA, Fulbright RK, Brown PB, McIntyre S, Nixon TW, Rothman DL, de Graaf RA. Deuterium metabolic imaging (DMI) for MRI-based 3D mapping of metabolism in vivo. *Sci Adv*; 2018; 4: eaat7314. DOI: 10.1126/sciadv.aat7314.
5. RA de Graaf, MA Thomas, KL Behar, and HM De Feyter. Characterization of Kinetic Isotope Effects and Label Loss in Deuterium-Based Isotopic Labeling Studies. *ACS Chem. Neurosci*. 2021; 12: 234 – 234. https://dx.doi.org/10.1021/acschemneuro.0c00711
6. Lin A, Andronesi O, Bogner W, et al. Minimum Reporting Standards for in vivo Magnetic Resonance Spectroscopy (MRSinMRS): Experts' consensus recommendations. *NMR in Biomedicine*. 2021; 34:e4484. https://doi.org/10.1002/nbm.4484
